# Capmatinib is an effective treatment for MET-fusion driven pediatric high-grade glioma and synergizes with radiotherapy

**DOI:** 10.1186/s12943-024-02027-6

**Published:** 2024-06-07

**Authors:** Marc Zuckermann, Chen He, Jared Andrews, Aditi Bagchi, Roketa Sloan-Henry, Brandon Bianski, Jia Xie, Yingzhe Wang, Nathaniel Twarog, Arzu Onar-Thomas, Kati J. Ernst, Lei Yang, Yong Li, Xiaoyan Zhu, Jennifer K. Ocasio, Kaitlin M. Budd, James Dalton, Xiaoyu Li, Divyabharathi Chepyala, Junyuan Zhang, Ke Xu, Laura Hover, Jordan T. Roach, Kenneth Chun-Ho Chan, Nina Hofmann, Peter J. McKinnon, Stefan M. Pfister, Anang A. Shelat, Zoran Rankovic, Burgess B. Freeman, Jason Chiang, David T. W. Jones, Christopher L. Tinkle, Suzanne J. Baker

**Affiliations:** 1 Hopp Children’s Cancer Center Heidelberg (KiTZ), Heidelberg, Germany; 2 Division of Pediatric Neurooncology, German Cancer Research Center (DKFZ) and German Cancer Consortium (DKTK), Im Neuenheimer Feld 280, Heidelberg, Germany; 3 Division of Pediatric Glioma Research, German Cancer Research Center (DKFZ), Im Neuenheimer Feld 280, Heidelberg, Germany; 4 Department of Developmental Neurobiology, St. Jude Children’s Research Hospital, 262 Danny Thomas Place, Memphis, TN 38105 USA; 5 Department of Oncology, St. Jude Children’s Research Hospital, 262 Danny Thomas Place, Memphis, TN 38105 USA; 6 Center for Pediatric Neurological Disease Research, St. Jude Children’s Research Hospital, 262 Danny Thomas Place, Memphis, TN 38105 USA; 7 Department of Radiation Oncology, St. Jude Children’s Research Hospital, 262 Danny Thomas Place, Memphis, TN 38105 USA; 8 Preclinical Pharmacokinetics Shared Resource, St. Jude Children’s Research Hospital, 262 Danny Thomas Place, Memphis, TN 38105 USA; 9 Department of Chemical Biology and Therapeutics, St. Jude Children’s Research Hospital, 262 Danny Thomas Place, Memphis, TN 38105 USA; 10 Department of Biostatistics, Departments of BiostatisticsSt. Jude Children’s Research Hospital, Memphis, 262 Danny Thomas Place, TN 38105 USA; 11 Department of Pathology, Departments of PathologySt. Jude Children’s Research Hospital, 262 Danny Thomas Place, Memphis, TN 38105 USA; 12 Center for Applied Bioinformatics, St. Jude Children’s Research Hospital, 262 Danny Thomas Place, Memphis, TN 38105 USA; 13 St. Jude Graduate School of Biomedical Sciences, St. Jude Children’s Research Hospital, 262 Danny Thomas Place, Memphis, TN 38105 USA; 14 Department of Pediatric Oncology, Hematology and Immunology, Heidelberg University Hospital, Im Neuenheimer Feld 400, 69120 Heidelberg, Germany; 15 Faculty of Biosciences, Heidelberg University, Heidelberg, Germany; 16 Center Of Excellence in Neuro-Oncology Sciences, St. Jude Children’s Research Hospital, 262 Danny Thomas Place, Memphis, TN 38105 USA

**Keywords:** Combination therapy, Pediatric-type diffuse high-grade glioma, Radiosensitization, MET inhibition, Preclinical trials, Capmatinib

## Abstract

**Background:**

Pediatric-type diffuse high-grade glioma (pHGG) is the most frequent malignant brain tumor in children and can be subclassified into multiple entities. Fusion genes activating the MET receptor tyrosine kinase often occur in infant-type hemispheric glioma (IHG) but also in other pHGG and are associated with devastating morbidity and mortality.

**Methods:**

To identify new treatment options, we established and characterized two novel orthotopic mouse models harboring distinct MET fusions. These included an immunocompetent, murine allograft model and patient-derived orthotopic xenografts (PDOX) from a MET-fusion IHG patient who failed conventional therapy and targeted therapy with cabozantinib. With these models, we analyzed the efficacy and pharmacokinetic properties of three MET inhibitors, capmatinib, crizotinib and cabozantinib, alone or combined with radiotherapy.

**Results:**

Capmatinib showed superior brain pharmacokinetic properties and greater *in vitro* and *in vivo* efficacy than cabozantinib or crizotinib in both models. The PDOX models recapitulated the poor efficacy of cabozantinib experienced by the patient. In contrast, capmatinib extended survival and induced long-term progression-free survival when combined with radiotherapy in two complementary mouse models. Capmatinib treatment increased radiation-induced DNA double-strand breaks and delayed their repair.

**Conclusions:**

We comprehensively investigated the combination of MET inhibition and radiotherapy as a novel treatment option for MET-driven pHGG. Our seminal preclinical data package includes pharmacokinetic characterization, recapitulation of clinical outcomes, coinciding results from multiple complementing *in vivo* studies, and insights into molecular mechanism underlying increased efficacy. Taken together, we demonstrate the groundbreaking efficacy of capmatinib and radiation as a highly promising concept for future clinical trials.

**Supplementary Information:**

The online version contains supplementary material available at 10.1186/s12943-024-02027-6.

## Background

Brain tumors are the leading cause of cancer-related death in children, with pediatric-type diffuse high-grade gliomas (pHGG) being one of the most aggressive tumor families [1]. Patients suffering from pHGG are typically treated with tumor resection followed by chemotherapy and/or radiation (based on age at diagnosis). This therapy is rarely curative and results in a 5-year survival rate of only ~20% [2]. Oncogenic fusions with receptor tyrosine kinase (RTK) genes *NTRK, ALK, ROS* or *MET* drive a subgroup of pHGG in infants (IHG, Infant-type hemispheric glioma) [3–6]. IHG has better survival than other pHGG[3, 4], but poses a significant therapeutic challenge and is associated with devastating long-term sequelae [5]. In pHGG patients >3 years old, *MET* fusions occur in up to 12 % of cases [6–8], and have also been identified in up to 15% of secondary glioblastoma in adults[9]. Recent advances yielded remarkable responses of *NTRK* or *ALK* fusion pHGG to selective inhibitors [10, 11], especially in IHG, but there is currently no effective selective therapy demonstrated for *MET* fusion-positive glioma.

A plethora of studies have explored new treatment options for pHGG, with solely discouraging outcomes [12]. Although novel small molecule inhibitors frequently show promising initial responses, a decade of experience has shown that monotherapy of pHGG inevitably results in therapy-resistant relapses [13]. The first FDA-approved inhibitor to target MET was crizotinib (Xalkori®). In the context of brain tumors, crizotinib displayed initial efficacy in a patient with pHGG [7], unfortunately followed by rapid progression. Capmatinib, another highly specific MET inhibitor, has shown promising intracranial activity [14, 15]. However, capmatinib has not been investigated as a treatment option against pHGG so far.

Given the limitations of monotherapies, multiple studies have investigated radiosensitization of tumor cells through RTK inhibition [16]. These included MET inhibitors, whose radiosensitizing effects were reportedly mediated by downregulation of DNA repair genes including *ATM* and/ or by anti-apoptotic factors [17–19]. However, the effect seems to be model-, tumor- and inhibitor-dependent [20]. So far, MET inhibition-mediated radiosensitization has not been explored in the context of pediatric brain tumors.

## Methods

All methods and materials are described in the Supplementary Methods (Additional File 1).

## Results

### Clinical presentation

**Fig. 1:**
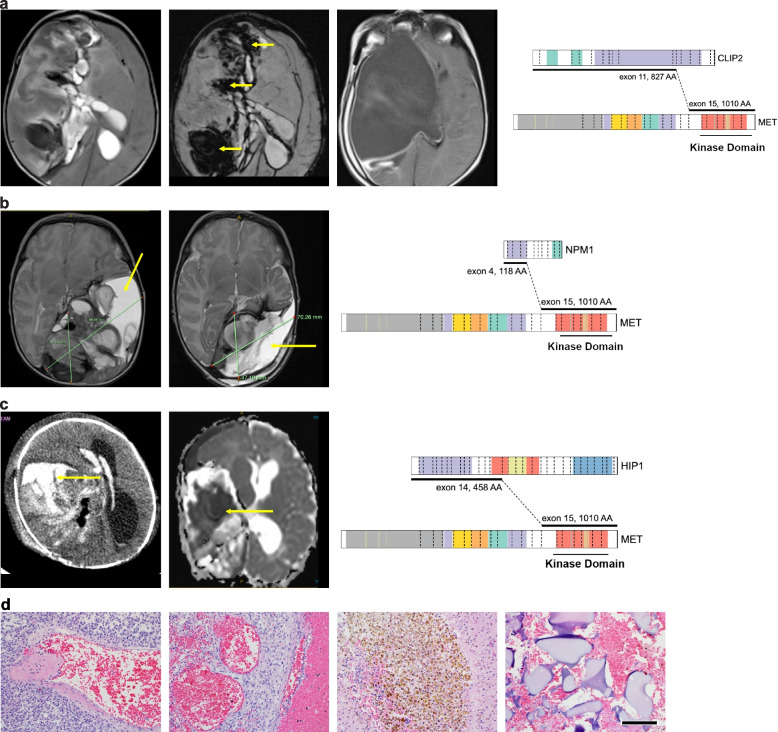
MET fusion IHG are large vascular tumors posing significant surgical challenges. **a** MRI images of IHG with CLIP2-MET fusion (right panel). Left panel: Left: T2 weighted image shows a large solid cystic tumor encompassing the entire right cerebral hemisphere, Middle: Subtraction weighted Image sequences (SWI). The yellow arrows indicate intra tumoral hemorrhagic regions. Right: T2 weighted image shows large tumor resection cavity after surgery. **b** MRI images of IHG with NPM1-MET fusion (right panel). Left panel: Left: T2 weighted MRI Image shows a large solid cystic tumor encompassing the entire temporal lobe of the left hemisphere. Right: Image post first attempt neuro-surgical resection. Due to massive bleeding and hemorrhage during surgery only a fraction of tumor could be resected. The yellow arrows show the large cysts within the tumor. **c** Images of IHG with HIP1-MET fusion (right panel). Left panel: Left: An emergent CT scan performed in the ER on a 4-week-old baby who presented with irritability and bulging anterior fontanelle. Shows a massive right hemispheric hemorrhagic tumor. The yellow arrow points toward the large hemorrhagic focus. Right: Diffusion Restricted images (DWI) of MRI. The restricted water diffusion (dark/black area noted by yellow arrow) represents high cellular density and proliferating tumor. **d** Histologic sections of a human MET-fusion tumor (TRIM24::MET) show large and abnormal thin-walled vessels invaded by the tumor cells (both upper panels), with mural thrombi (two left panels) and acute hemorrhages (second from left). Large areas of hemosiderin deposition, evidence of prior hemorrhages and hematoma, are noted in the tumor (second from right). Ample amounts of Gelfoam were needed to achieve hemostasis during surgery (far right). Scale bar is 150µm

We analyzed MRI scans from MET fusion IHG patients enrolled on the SJYC07 clinical trial (NCT00602667) [5] or standard institutional protocols, which illustrated typical challenges for IHG surgery. The tumors are often very large, vascular and hemorrhagic, and associated with intraoperative bleeding, difficulties achieving gross total resections, and high morbidity (Fig. 1a-d). Fusion events between CLIP2 and MET have been observed in IHG and pHGG before [3, 7], whereas, to our knowledge, we are the first to identify NPM1 and HIP1 as alternative fusion partners of MET. Our institutional experience thus confirmed the significant clinical challenges for MET fusion pHGG patients and the need for novel therapeutic concepts.

### A novel, immunocompetent mouse model for MET-driven pHGG

**Fig. 2:**
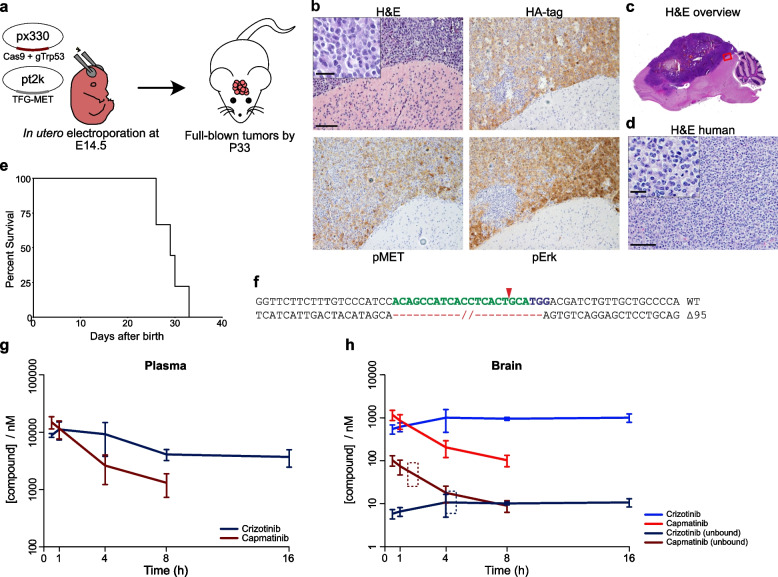
TFG-MET-driven mouse model and pharmacokinetic profiles of MET inhibitors. **a** Schematic illustrating the method and utilized vectors to induce CRISPR/Cas9-mediated *Trp53* deletion and *TFG-MET* overexpression following *in utero* electroporations. **b** H&E staining and Immunohistochemical analysis of a tumor generated by *in utero* electroporation, visualized by the HA-tag of TFG-MET. In contrast to normal tissue (bottom right corners) tumors display elevated levels of pMET and pErk. Scale bars are 100 µm in large panel and 25 µm in high magnification inset. **c**, H&E staining showing a large and invasive HGG in the mouse brain. Red rectangle indicates the region shown in b. **d** H&E staining of a human MET-driven pHGG demonstrating similar features as murine neoplasms. Scale bars are 100 µm in large panel and 25 µm in high magnification inset. **e** Survival curve indicating penetrance and latency of tumors induces by *in utero* electroporation. **f,** Sanger sequencing of PCR products of the targeted *Trp53* locus in a tumor revealed a 95bp deletion in all analyzed sequences (*n*=6). **g, h** Plasma(G)- and brain(H)-concentrations of capmatinib and crizotinib at the indicated time points after administration of CD-1 nude mice with the respective compounds. Three mice were analyzed per compound and time point. Error bars indicate the standard deviation. Dashed rectangles indicate time windows of radiation in the following preclinical allograft study

To initially develop a genetically defined model of the disease, we performed *in utero* electroporation to stably induce expression of the HA-tagged, human *TFG-MET* fusion gene as well as a CRISPR-mediated knockout of *Trp53* in the forebrain of E14.5 mice (Fig. 2a). We chose TFG-MET because it is the smallest identified fusion in both, IHG and pHGG[3, 7] (Supplementary Fig.1a; Additional File 2), fostering efficient somatic gene delivery. All electroporated mice developed tumors that stained positive for the HA-tag, pMET and pErk, validating the delivered fusion gene as an oncogenic driver (Fig. 2b,c). Murine tumors (Fig. 2b) showed similar histopathology to human, MET-fusion driven HGG (Fig. 2d), including characteristically round and relatively monotonous morphology as well as cytoplasmic clearing (Fig. 2b,d, higher magnification boxes, Supplementary Fig. 1b; Additional File 2). Our electroporation model was robust and highly aggressive with 9/9 mice developing neurologic symptoms by day 33 after birth (Fig. 2e). We showed that *Trp53* knockout was efficient, inducing a 95 base pair deletion in all analyzed clones (Fig. 2f *n*=6), thereby recapitulating the loss of *TP53* function that is frequently observed in patients with *MET*-activated pHGG [7]. The results highlight a novel mouse model with short latency and full penetrance that reflects the histopathology of the human counterpart.

### Capmatinib demonstrates a favorable PK profile in mice compared to crizotinib

To evaluate the brain exposure of crizotinib and capmatinib, we analyzed their PK profiles in CD1 nude mice. Capmatinib was rapidly absorbed and cleared from both, brain and plasma, with concentrations below the detection limit at 16 hours post-dose (Fig. 2g, h and Supplementary Table 1; Additional File 3) while crizotinib slowly equilibrated, reaching Cmax at 4 hours post dose. Both drugs, however, reached physiologically relevant concentrations of >1 µM in brain tissues. Previous studies showed that unbound drug concentrations predict target inhibition more robustly than total amounts [21]. Therefore, we performed *in vitro* protein binding assays with standard mouse plasma and naive brain tissue homogenates, finding that capmatinib has an appreciably higher fraction unbound in brain homogenate (Fu,b) versus crizotinib (Table 1). We then used respective Fu,b values to estimate unbound concentrations (Fig. 2h) and found that capmatinib reaches a 9.6-times higher maximal concentration of unbound drug in the brain than crizotinib (~103nM vs ~11nM).

**Table 1:** *In vitro* ADME (adsorption, distribution, metabolism, and excretion) profiling of crizotinib, capmatinib and cabozantinib. AVG = average, STD = standard deviation. Values indicate the unbound drug fractions in the depicted environment

*Drug*	*Mouse Plasma*		*Mouse Brain Tissue*		*Culture Media*	
	AVG	STD	AVG	STD	AVG	STD
*Crizotinib*	2.23%	0.19%	1.06%	0.08%	61.65%	2.95%
*Capmatinib*	5.02%	0.47%	11.25%	5.81%	70.49%	2.45%
*Cabozantinib*	0.17%	0.03%	0.25%	0.03%	3.30%	0.26%

### Capmatinib efficiently inhibits TFG-MET in vitro and in vivo

**Fig. 3:**
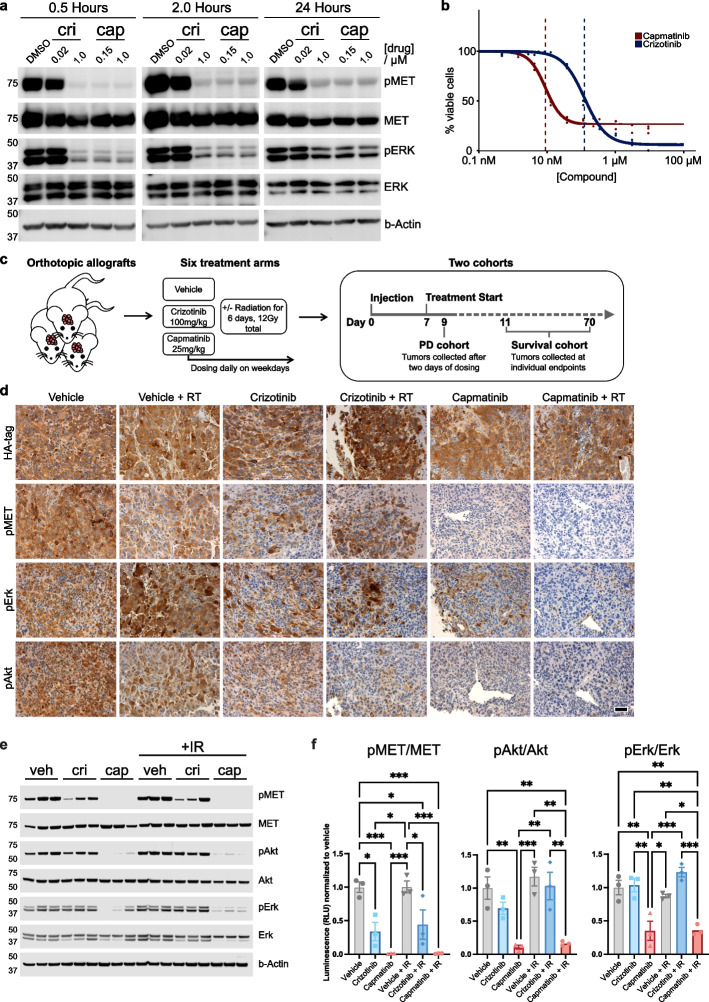
Capmatinib is effective against TFG-MET-driven tumor cells. **a** Western blot of phosphorylated and total MET and the downstream effector Erk in cultured, murine tumor cells after different time points of crizotinib (cri) or capmatinib (cap) addition at the indicated concentrations. **b** Dose-response curves of murine tumor cells after treatment with capmatinib or crizotinib. Each dot represents one replicate of triplicates. Viable cells were analyzed 72 hours after compound addition using the CellTiter-Glo Assay. The vertical dotted lines indicate EC50 values. **c** Overview schematic depicting the various treatments and the two different cohorts of our preclinical allograft study. **d** Immunohistochemical stainings of phosphoproteins in tumors of the PD cohort, which were treated with the indicated therapies. Levels of pMET, pErk and pAkt were significantly reduced after capmatinib treatment. Scale bar is 50 µm. **e** Western blot of phosphorylated and total MET, Akt and Erk from allograft tumors treated with vehicle (veh), crizotinib or capmatinib alone or in combination with irradiation. **f** Quantification of luminescence signal of western blots in panel C normalized to the respective vehicle control. Each dot represents an individual replicate. Error bars display standard error of the mean. Statistical significance was determined using a One-Way ANOVA followed by Tukey’s multiple comparisons test (*p<0.05, **p<0.01, ***p<0.001, ****p<0.0001)

We cultured tumor cells from our electroporation model *in vitro* and analyzed the impact of crizotinib or capmatinib treatment on phosphorylation of MET and downstream effectors (Fig. 2a). To investigate the intracellular response at relevant *in vivo*-concentrations, we challenged the cells with Cmax equivalents, based on the identified, free drug concentrations in the murine brain (0.02 µM of crizotinib and 0.15 µM of capmatinib; Fig. 2h). For both compounds 1 µM was used as positive control. Capmatinib readily inhibited the phosphorylation of TFG-MET and downstream targets Erk and Akt at both tested concentrations, while the Cmax equivalent dose of crizotinib displayed minimal effect (Fig. 3a and Supplementary Fig. 1c; Additional File 2). A dose response assay similarly revealed an *in vitro* potency of capmatinib >10 times higher than that of crizotinib (Fig. 3b and Supplementary Table 2; Additional File 4). Of note, capmatinib readily inhibited MET, Erk and Akt phosphorylation at the observed *in vitro* IC_50_ concentration of only 9 nM (Supplementary Fig. 1d,e; Additional File 2), further emphasizing its potency. Next, we combined both compounds with RT and observed an increased anti-tumoral efficacy compared to single treatments (Extended Data Fig. 1f,g). Both combinations have an additive effect with a trend towards synergy according to the ZIP synergy model[22], with some clearly synergistic dose ranges (Supplementary Fig. 1h; Additional File 2). The capmatinib synergy score peaked at ~100 nM, an achievable free drug concentration in the murine brain (Fig. 2h).

To analyze the efficacy of capmatinib and crizotinib *in vivo*, we allografted tumor cells from our electroporation model into CD1 mice, providing a standardized mouse model with an immunocompetent background. In order to utilize this model in a combinatorial RT trial, we first determined a radiation dose at which allografted mice developed a partial but not a full response. As an initial study using 20 Gy demonstrated complete tumor remission in two out of five treated mice (Supplementary Fig. 2a,b; Additional File 5), we lowered the total dose to 12 Gy (in typical clinical fractions of 2 Gy per day [23]) for the subsequent, combinatorial trial in which animals received either A) vehicle B) vehicle + RT C) crizotinib D) crizotinib + RT E) capmatinib F) capmatinib + RT (Fig. 3c). All regimens were well tolerated (Supplementary Fig. 2c; Additional File 5). The time point of radiation was chosen to coincide with the Cmax of the respective drug in the brain (Fig. 2h, dashed squares). To analyze pharmacodynamic properties, mice were sacrificed after receiving their second treatment. These animals formed the “PD cohort” whereas the remaining mice represented the “Survival cohort”. Treatment with capmatinib led to greatly reduced levels of phosphorylated MET, Erk and Akt in initial neoplasms of PD animals, whereas crizotinib treatment induced a less complete reduction compared to vehicle-treated mice (Fig. 3d,e and f and Supplementary Fig. 3; Additional File 6). These results indicate that capmatinib readily inhibits MET in intracranial tumors at clinically relevant doses.

### Combined capmatinib and RT increases survival-rate and -time of murine allografts

**Fig. 4:**
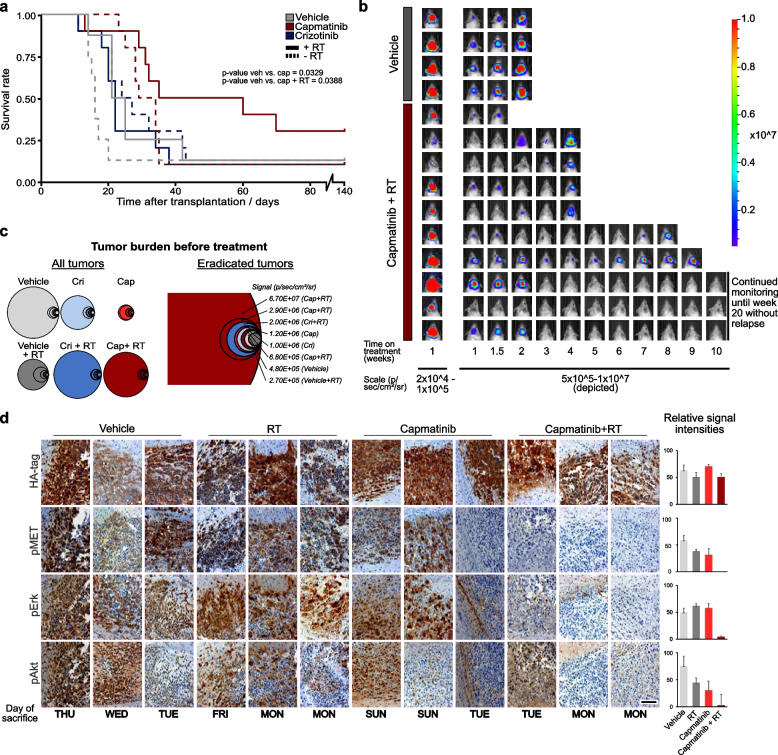
Combining capmatinib and RT increases survival-rate and -time *in vivo*. **a** Kaplan–Meier curve of mice enrolled in the “Survival cohort”. All treatments started 1 week after transplantation. Radio therapy was administered for 6 days, delivering 12 Gy total. Compound treatment was continued for 84 days. After an additional 49 days of monitoring (140 days after transplantation) the trial ended and none of the remaining mice showed any hints of residual tumor. Three mice that were treated with capmatinib + RT reached this time point, whereas each of the other groups contained only 1 “survivor”. N = 8 (vehicle arms) or n = 10 (compound-treated arms), respectively. *P*-values for groups that displayed statistically significant survival differences are indicated. **b** Bioluminescence-imaging pictures from four representative mice of the vehicle arm (middle ranks according to initial luciferase intensity) and from all mice of the capmatinib + RT arm. First row is depicted in another intensity scale to visualize tumors in all mice. The depicted scale bar indicates the range from 5x10^5-1x10^7 photons/sec/cm^2^/sr. The combinatorial treatment induced tumor regression in 8/10 animals around day 21 on treatment. **c** Tumor burdens according to BLI of all enrolled mice before treatment are depicted as area of circles (left panel). The right panel shows the initial tumor sizes of mice that survived for 140 days without residual tumor. While the surviving animals of the vehicle groups displayed the smallest initial tumors, neoplasms of all sizes could be cured with combinatorial therapy of capmatinib and radiation. **d** Immunohistochemical analyzes of phosphoproteins in tumors of the Survival cohort, which were treated with the indicated therapies until onset of neurological symptoms. Phospho-MET, pErk and pAkt levels were significantly reduced in capmatinib-treated mice collected on days of treatment (Mo.-Fr.), however elevated levels reappeared in tissue collected during treatment pauses on weekends. Scale bar is 100µm

We monitored the mice of the Survival cohort for up to 140 days after transplantation. All treatments increased the average survival time compared to the vehicle treated group, which was most prominent for animals treated with capmatinib + RT (Fig. 4a; *p*-value vehicle vs. capmatinib + RT=0.0388). Besides the prolonged duration of survival, this combination also increased the survival rate 3-fold (Fig. 4a). Additionally, biweekly bioluminescence imaging allowed us to quantify the combinatorial effect of capmatinib + RT (Fig. 4b, Supplementary Table 3; Additional File 7). While all other treatments mostly slowed down tumor growth, 8/10 capmatinib + RT treated animals displayed a reduction of tumor burden by week 3 (Fig. 3b, Supplementary Fig. 2d and Supplementary Table 3; Additional Files 5 and 7). Capmatinib and RT combined was able to eradicate even large initial neoplasms whereas the survivors in other groups were mice with low initial tumor burden (Fig. 4c).

Brains of mice that had to be sacrificed under treatment were histologically analyzed (Fig. 4d). As expected, mice treated with vehicle or radiation alone showed a strong upregulation of pMET, pErk and pAkt. Interestingly, the amounts of pMET and pErk were reduced to background levels in only 4/6 capmatinib-treated animals. This reflected the time span between last capmatinib administration and tumor isolation, as the 2 mice with stronger pMET/ pErk signal were sacrificed after a 2-day treatment pause, underscoring the observed rapid clearance of capmatinib in the brain (Fig. 2h).

### Capmatinib effectively inhibits TRIM24-MET in human pHGG

**Fig. 5.**
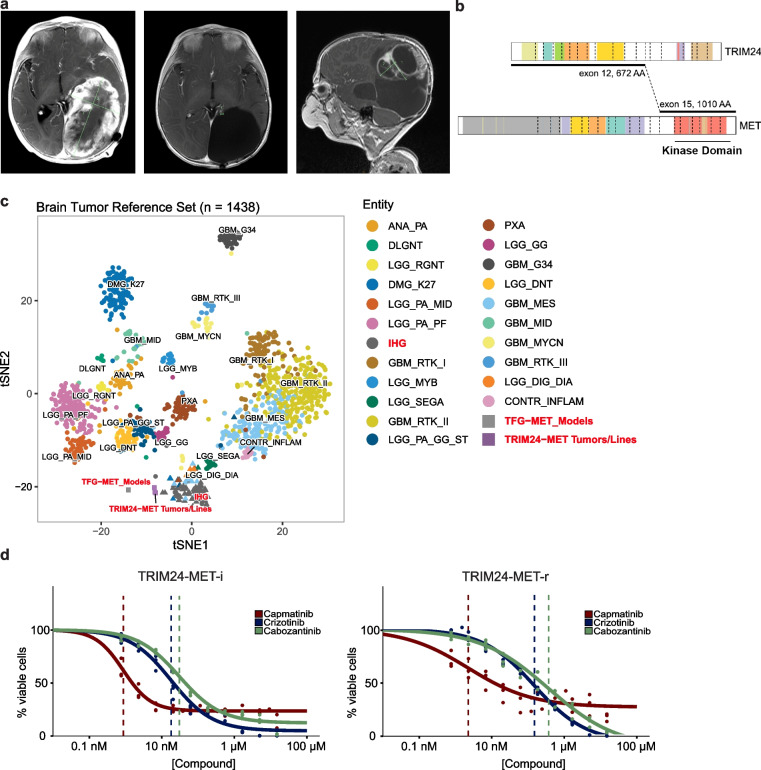
Sensitivity of human tumor samples to MET inhibition. **a** MRI images from an IHG patient with TRIM24-MET fusion. Left panel: Image at diagnosis showing a large solid cystic tumor filling the entire temporal lobe of the left hemisphere. Middle panel: Image at the end of resection and chemotherapy. Right panel: MRI image at recurrence. **b** The fusion encompassed *TRIM24* exons 1-12 and exon 15 of *c-MET*, encoding a chimeric protein that contains the N-terminal moiety of TRIM24 and the c-MET kinase domain. **c,** tSNE projection of a combined methylation dataset comprised of a reference set of glioma subtypes (n=1128, circles from Capper, et al. Nature 2018, triangles from Clarke, et al. Cancer Discov 2020). The TRIM24-MET and TFG-MET tumor samples and cell lines from this study (squares, TRIM24-MET-i primary *n* = 4, cell culture *n* = 1; TRIM24-MET-r primary *n* = 3, cell culture *n* = 1; TFG-MET Models *n* = 6) group together with infant HGG with RTK fusion genes (IHG). **d** Dose-response curves of TRIM24-MET-i and TRIM24-MET-r cells after treatment with capmatinib, crizotinib or cabozantinib for 72 h. Data from three independent experiments. The vertical dotted lines indicate EC50 values

During the time of this study a seven-month-old infant presented with a large cerebral mass and leptomeningeal metastasis extending from the brainstem through the cervical spine (C1-7; Fig. 5a). After surgical resection of the cerebral tumor, the patient received six months of chemotherapy, as the patient was deemed too young for radiation therapy post-surgery. The patient had no evidence of disease at the end of therapy (Fig. 5a) but relapsed within seven months thereafter. Molecular analysis revealed a TRIM24-MET fusion in both the initial and the recurrent tumors (Fig. 5b), however subsequent treatment with the MET inhibitor cabozantinib was ineffective.

Samples of the pre-treatment (TRIM24-MET-i) and recurrent tumor (after chemotherapy but before cabozantinib; TRIM24-MET-r) were obtained for further characterization and disease modelling (Supplementary Fig. 4a-e; Additional File 8). The two samples were used to establish two stably growing cell cultures and expression of the fusion (predicted molecular weight of 116.8 Kd) was validated by immunoprecipitation (Supplementary Fig. 4f-h; Additional File 8). We performed DNA methylation profiling of both primary biopsies and the corresponding established cultures and found that all samples cluster closely with RTK fusion-driven IHG. We also profiled six biological replicates of our murine TFG-MET tumors using MM285k arrays, performed a cross-species implementation and found that the murine tumors clustered closely to human IHG as well (Fig. 5c).

We challenged human tumor cells with brain-specific unbound Cmax equivalents of capmatinib and crizotinib. Both drugs inhibited phosphorylation of TRIM24-MET and ERK within 30 minutes (Supplementary Fig. 5a; Additional File 9). In comparison to murine tumor cells, the Cmax-equivalent dose of crizotinib also resulted in an observable inhibition, albeit to a lesser extent than 1 µM crizotinib or any analyzed capmatinib concentration (Supplementary Fig. 5b; Additional File 9). In dose-response assays, we found that capmatinib was more potent than crizotinib and cabozantinib (Fig. 5d), similar to our observations in murine tumor cells. To validate capmatinib’s potency in additional MET-fusion-driven pHGG models, we also performed dose response assays with SJ-GBM2 cells [24], harboring a CLIP2*-MET* fusion and with cells isolated from murine tumors, induced by overexpressing *TFG-MET* alone (without *Trp53* knockout; Supplementary Fig. 5c,d; Additional File 9). Capmatinib potently inhibited both models and displayed an IC_50_ of only ~1.17nM against SJ-GBM2 cells, further underscoring its effectiveness against pHGG driven by MET-fusions.

### Capmatinib treatment leads to long-term progression-free survival of human xenografts

To investigate capmatinib's anti-tumor efficacy on human cells *in vivo*, we established a novel PDOX model using TRIM24-MET-i cells. Given the observed rapid clearance of capmatinib in mouse tissues and tumors (Fig. 1h and 3d), we chose to administer capmatinib twice per day (*bis in die*, BID) to PDOX mice, matching the clinical dosing schedule [25]. Subsequent western blot and IHC analysis revealed that capmatinib efficiently blocked phosphorylation of TRIM24-MET, ERK and AKT on this schedule (Supplementary Fig. 6a,b; Additional File 10).

**Fig. 6:**
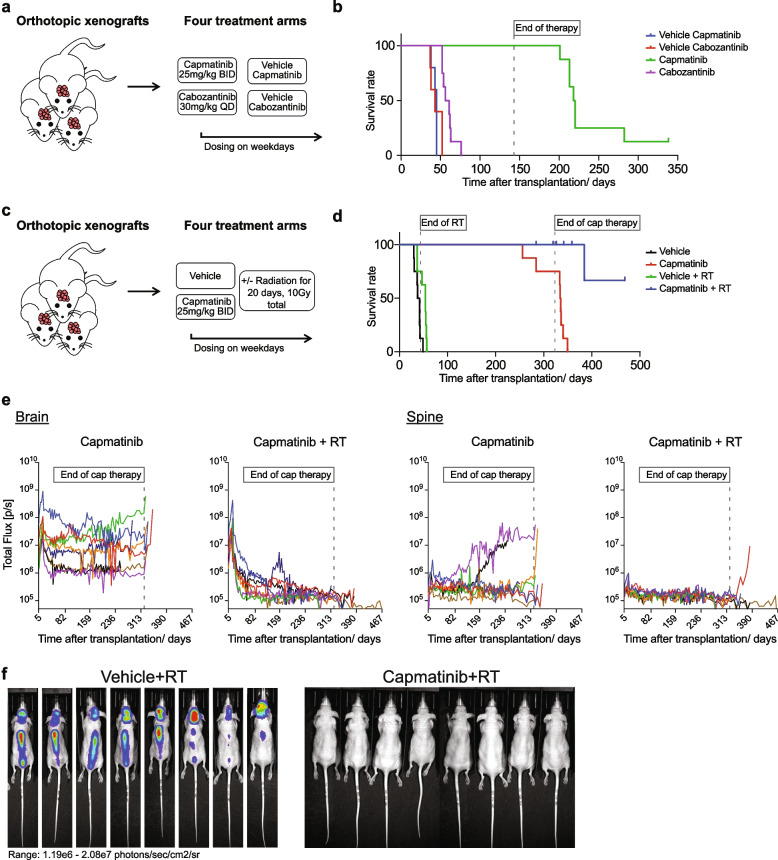
Combination of capmatinib and RT eradicates human tumor cells *in vivo. ***a** Overview schematic depicting the four treatment arms of the preclinical study comparing *in vivo* response to capmatinib and cabozantinib. **b** Kaplan–Meier curve of mice enrolled in the study depicted in A. All treatments started 13 days after transplantation. Compound treatment was continued for 133 days. Within the subsequent 6 months of monitoring, 7 of 8 mice in the capmatinib-treated group experienced tumor relapse. **c** Overview schematic depicting the four treatment arms of the preclinical study comparing the combination treatment of capmatinib and RT *vs* either treatment alone. **d** Kaplan–Meier curve of mice enrolled in the study depicted in C. All treatments started 18 days after transplantation. Radiotherapy was administered at 0.5 Gy per day, delivering 10 Gy total. Compound treatment was continued for 301 days. After an additional 147 of monitoring, the trial ended with all mice having reached their tumor-induced or natural endpoint. **e** Trend of total flux (photons/sec/cm2/sr) at the cranial and spinal cord region of capmatinib-treated mice enrolled in the 4-arm preclinical trial depicted in C. **f** Bioluminescence-imaging pictures from mice of the vehicle + RT arm at the time closest to the humane endpoint and from capmatinib + RT treated mice at that time. Color scale range: 1.19x10^6-2.08x10^7 photons/sec/cm^2^/sr

To determine how closely our PDOX model would recapitulate the clinical failure of cabozantinib, we directly compared capmatinib vs. cabozantinib treatment (Fig 6a). Cabozantinib treatment resulted in a 15.5 day increase of median survival (p-value cabozantinib vs. cabozantinib vehicle = 0.002). Despite the statistical significance, this slight reduction of tumor growth would likely not have been appreciable clinically and is therefore consistent with the lack of efficacy in the patient. In striking contrast, capmatinib induced a long-term stable disease with all mice surviving the 19-week treatment period (Fig 6b; p-value capmatinib vs. capmatinib vehicle < 0.0005). Regular luciferase imaging underscored the long-term tumor control and even indicated initial regression in two out of eight capmatinib-treated mice (Supplementary Fig. 6c; Additional File 10). Ultimately seven of these animals relapsed after treatment was ceased (Fig. 6b), indicating that capmatinib monotherapy is not sufficient to consistently induce complete remission.

Consequently, we combined capmatinib with RT in human cells and found that radiation increased the response to capmatinib treatment *in vitro* (Supplementary Fig. 6d; Additional File 10). We then conducted a 4-arm preclinical trial treating the TRIM24-MET-i PDOX model with: 1) vehicle, 2) vehicle+RT, 3) capmatinib, and 4) capmatinib+RT (Fig. 6c). As MET-fusion-driven tumors are often diagnosed in infants [3] and we aimed to extend the time frame of potential synergy whereby cells were exposed to both capmatinib and RT and chose a very low-dose fractionation [26] of 0.5 Gy per day, with a total dose of 10 Gy over 20 days to recapitulate a clinical scenario balancing risk and benefit in pediatric patients. All treatments were well tolerated (Supplementary Fig. 6e; Additional File 10). RT alone resulted in a slight survival benefit compared to vehicle-treated mice (Fig. 6d,e, 7.7 weeks vs 5.7 weeks, Log-rank test p=0.0086). Beside their cranial tumor outgrowth, all mice in these two groups quickly developed spinal metastases (Supplementary Fig. 6f; Additional File 10). Capmatinib monotherapy again induced a stable disease in all treated animals but persistent tumor cells in both, brains and spines readily grew out once treatment was withdrawn (Fig. 6d,e). Thereby, all mice treated with capmatinib or RT alone eventually reached tumor-induced endpoints. In striking contrast, combined capmatinib + RT profoundly and stably decreased tumor burden (Fig. 6d,e and f). Importantly, although radiation was focally administered to the head, only one capmatinib + RT treated mouse experienced a spinal metastasis after therapy was withdrawn, while the remaining mice did not show any detectable signs of residual tumor before reaching natural, cancer-independent endpoints (Fig. 6e,f). Taken together, these results show that also in the context of a human-derived MET-driven pHGG model, only the combination of capmatinib and RT reduces tumor burden and leads to long-term, progression- and metastasis-free survival.

### Capmatinib induces dysregulation of DNA repair genes as a possible mechanism of radiosensitizaton

**Fig. 7:**
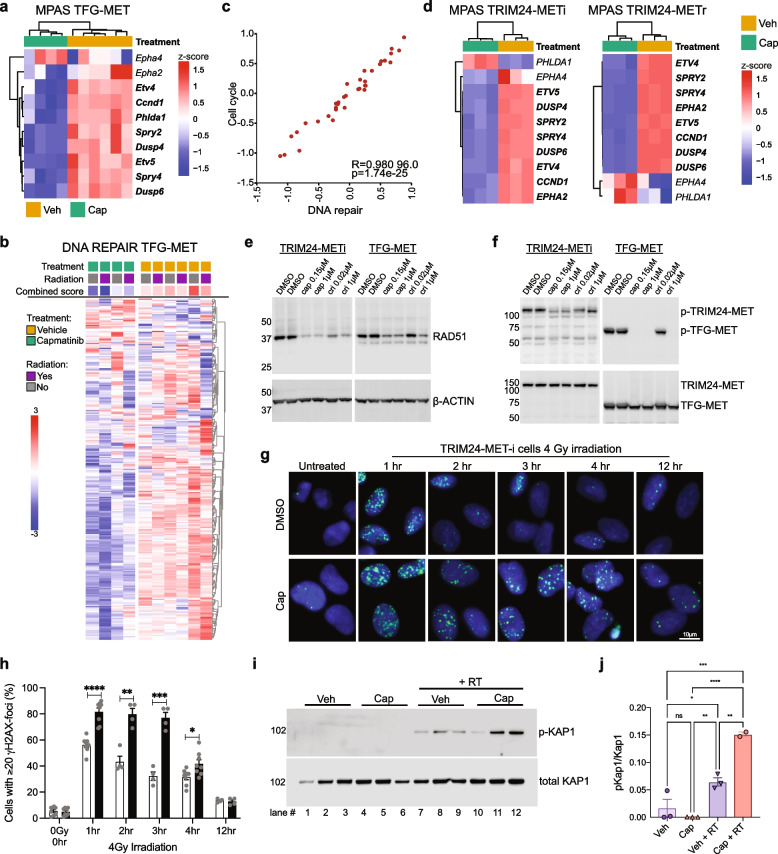
Capmatinib dysregulates expression of DNA repair genes and enhances radiation-induced DNA damage. **a** Expression of the indicated Mapk pathway signature (MPAS) genes in tumors of the PD cohort treated with vehicle (+/-RT) or capmatinib (+/- RT, focusing on the 4 strongly affected tumors by capmatinib-treatment, the 2 outliers were excluded for this analysis). With the exception of Epha4, expression of all analyzed Mapk pathway signature genes were inhibited in capmatinib-treated mice. Significantly downregulated (adj. *p* < 0.05) genes are in bold. **b** Heatmap of genes in the “DNA REPAIR_7” geneset (baderlab pathways 2019) demonstrating that capmatinib treatment leads to a reduced expression of DNA repair genes. **c** Correlation between total expression scores of the genesets “DNA REPAIR_7” and “CELL CYCLE_7” (baderlab pathways 2019) amongst all murine tumors (treated and untreated) analyzed by RNAseq in this study. Each dot represents one tumor. **d** Heatmaps showing expression of MAPK Pathway Activity Score (MPAS) genes for *in vitro* capmatinib treatments in cell lines derived from TRIM24-MET fusion tumors as compared to a DMSO vehicle control. Significantly downregulated (adj. *p* < 0.05) genes are in bold. **e** Western blots of RAD51 and β-ACTIN after indicated treatments of TRIM24-MET or TFG-MET cells for 24 hours. Capmatinib and crizotinib both induce downregulation of RAD51. **f** Western blots of MET and p-MET after indicated treatments of TRIM24-MET or TFG-MET cells for 24 hours, which serve as controls for Western Blots in e. **g** γH2AX-immunofluorescence staining of TRIM24-MET L97 human glioma cell lines at different recovery timepoints following 4 Gy-irradiation. Capmatinib (Cap)-treated cells display significantly higher levels of γH2AX compared to DMSO-treated (DMSO) cells. **h** quantification of γH2AX-foci in f. The percentage of cells with ≥20 γH2AX-foci is significantly higher in capmatinib-treated cells (black bar) compared to DMSO-treated cells (white bar) at 1, 2, 3 and 4 hours following irradiation. Error bars display standard error of mean, statistical significance was determined using t-test analysis. (****;*p*<0.0001, ***;*p*<0.001, **;*p*<0.01, *;*p*<0.05). Scale bar is 10µm. **i** Western blot of phosphorylated and total Kap1 from TFG-MET allograft tumors treated with vehicle (Veh), or capmatinib (Cap) alone or in combination with irradiation (RT). Samples 7-9 and 11-12 were collected 1 hr after RT, lane 10 was collected 3 hrs after RT, and shows time-dependent decrease of the DNA double-strand break signal. **j** Quantification of luminescence signal of western blots in panel h normalized to the vehicle control. Each dot represents an individual replicate. Error bars display standard error of the mean. Statistical significance was determined using a One-Way ANOVA followed by Tukey’s multiple comparisons test (*p<0.05, **p<0.01, ***p<0.001, ****p<0.0001). Lysate from lane 10 was excluded due to the different timepoint after RT

To investigate the molecular basis for the combined effect between capmatinib and RT, we performed RNA-sequencing analysis on murine tumors and validated fusion gene expression as well as p53 inactivation through frameshift in analyzed allografts (Supplementary Fig. 7a-d; Additional File 11). When analyzing the expression of *Mapk* signature genes [27], we found a significant downregulation in capmatinib treated tumors, whereas crizotinib-treated samples displayed a more heterogenous expression (Supplementary Fig. 7e; Additional File 11). To elucidate capmatinib’s molecular effect on the cells, we focused on the differentially expressed genes between 4 capmatinib-treated tumors showing a particularly strong *Mapk* downregulation (Fig. 7a) and the 6 vehicle-treated PD samples. As expected, we observed a downregulation of gene sets pertaining to proliferation pathways in capmatinib-treated mice (Supplementary Fig. 7f; Additional File 11). Capmatinib-treated tumors of the Survival cohort displayed more heterogenous gene expression patterns than the PD cohort, potentially owing to more variable responses to long term drug exposure (Supplementary Fig. 8a; Additional File 12). Importantly, genes involved in the DNA repair machinery were downregulated in capmatinib-treated tumors (Fig. 7b, Supplementary Fig. 8b and Supplementary Table 4; Additional Files 12 and 13), providing a plausible explanation for the radiosensitizing effect of this drug. In tumors of the Survival cohort, genes involved in cell cycle progression were found to be upregulated after radiation (Supplementary Fig. 8c; Additional File 12), potentially as a late consequence to radiation induced DNA damage and tumor cell selection. Consistent with this finding, we observed a strong correlation between upregulation of genes associated with increased proliferation and upregulation of genes associated with DNA repair across the entire cohort (Fig. 7c). Although the connection between proliferation and expression of DNA repair genes is well known, we found a striking correlation also in further analyzed datasets, including human brain tumors and cells of normal brain development (Supplementary Fig. 8d; Additional File 12), potentially indicating DNA repair gene dysregulation by cell cycle inhibition as general radiosensitization option for certain tumor entities. Furthermore, we found genes involved in *Trp53* regulation to be specifically downregulated in capmatinib-treated samples (Supplementary Fig. 8e,f; Additional File 12), which may contribute to the reduced expression of DNA repair genes despite the absence of *Trp53* itself in tumors (Supplementary Fig. 7b; Additional File 11).

To validate this finding in human cells, we also performed RNA-sequencing of TRIM24-MET-i and TRIM24-MET-r cells after 4h *in vitro* treatment with capmatinib, crizotinib, or cabozantinib. At their respective EC90 concentrations (Supplementary Table 5; Additional File 14), all three treatments caused similar transcriptional responses when compared to DMSO vehicle controls (Supplementary Fig. 9a and Supplementary Table 6; Additional Files 15 and 16). Downregulation of MAPK pathway signature genes confirmed successful MET inhibition (Fig. 7d). Next, we performed pre-ranked gene set enrichment analyses (GSEA) and found that cellular responses to capmatinib treatment between human tumor cultures and allografted mouse tumors were highly similar (Supplementary Tables 7, 8 and 9; Additional Files 17-19). Commonly downregulated genesets included MYC target genes, mTORC1 signaling, and unfolded protein response (Supplementary Fig. 9b,c; Additional File 15). In contrast to our murine tumors, we found that *TP53* is expressed in human tumor cells (Supplementary Table 6; Additional File 16). However, we also found a dysregulation of genes involved in DNA repair (Supplementary Fig. 10a; Additional File 20), similar to our observation in allograft models (Fig. 7b). A major DNA repair gene is *RAD51*, which is involved in DNA double strand break repair and frequently upregulated in various human cancers [28]. Despite no significant change in RNA levels, RAD51 protein is downregulated in both murine and human tumor cells after short-term *in vitro* drug treatment (Fig. 7e,f).

To further confirm the impact of capmatinib treatment on DNA repair, we treated cells with capmatinib or DMSO for 24 hours, performed irradiation and quantified γ-H2AX loci after an additional 1 to 24 hours. When treated with radiation alone, the number of γ-H2AX loci steadily declined over time in murine cells, indicating continuous DNA repair. The addition of capmatinib significantly impaired this process and prolonged recovery (Supplementary Fig. 10b,c; Additional File 20). In human tumor cells the effect was even more pronounced, as capmatinib treatment induced a greatly increased number of DNA double strand breaks (Fig. 7g,h). The ataxia telangiectasia mutated (ATM) kinase initiates a signaling cascade including phosphorylation of Kap1 (KRAB-associated protein 1) at serine 824 in response to DNA double strand breaks [29]. To further investigate the effects of capmatinib treatment on DNA damage response *in vivo*, we evaluated Kap1 pS824 in murine TFG-MET tumors*. *Phosphorylated Kap1 (p-Kap1), was dramatically increased in irradiated tumors treated with vehicle compared to unirradiated controls and showed a significantly greater increase in tumors treated with capmatinib and RT (Fig. 7i,j). Taken together these findings demonstrate that capmatinib treatment induces a dysregulation of DNA repair genes, and a marked potentiation of radiation-induced DNA damage *in vitro* and *in vivo*, providing a rational mechanism for the outstanding combinatorial efficacy in our animal models.

## Discussion

Activating alterations in receptor tyrosine kinases are appealing therapeutic targets that are increasingly identified by clinical genomic approaches, and often play important roles in tumor maintenance and survival. Despite a growing armamentarium of available selective RTK inhibitors, choosing the ideal drug and predicting successful tumor response is complicated by diverse factors [30]. While RTK inhibition displayed promising responses in multiple pHGG studies [31, 32], responsiveness of adult HGG to RTK inhibition proved to be less striking and is currently under investigation [33]. This discrepancy could partially result from the fact that pHGG, especially IHG, typically lacks large-scale structural, copy number, or single nucleotide variants [34, 35], rendering the tumor exclusively dependent on the oncogenic RTK such as MET. Targeting a MET fusion gene with crizotinib in one pHGG patient resulted in a partial response with rapid tumor relapse [7], yet no lasting response after MET inhibition has been demonstrated for MET-driven pHGGs so far. Neurosurgery for large vascular IHG is associated with high morbidity, such as intraoperative bleeding, hypovolemic shock, mechanical ventilation and permanent neurologic deficits. Attaining a gross total resection (GTR) is difficult, often requiring multiple craniotomies. Therefore, long-term survivors often suffer from permanent neurocognitive impairment, hemiparesis, seizure disorders, dysarthria, and visual deficits. In a recently published NEJM report, a patient was left moribund after two unsuccessful craniotomies to resect a large hemispheric tumor. As molecular analysis revealed an ALK fusion, the child was treated with ALK inhibitor on a palliative basis. Remarkably, the tumor showed rapid shrinking and could be safely surgically resected with good clinical recovery [11]. Similar cases have also been reported with NTRK fusion [10] pHGG. However, there is currently no effective selective inhibitor therapy for MET fusion-driven pHGG.

In this study, we established complementary *in vitro* and *in vivo* models of MET-driven pHGG including an immunocompetent allograft with TFG-MET fusion and *Trp53* deletion. In contrast to a previous RCAS TFG-MET-driven pHGG model [7], the allograft described here is studied in an immunocompetent, wild-type p53 host background and allows robust preclinical evaluation by standardized tumor cell transplantation. Additionally, we generated two patient-derived cell lines and matched xenografts with TRIM24-MET fusion. All of our models closely recapitulated patient primary tumors as demonstrated by histopathology and methylation profiling. They thereby allowed us to faithfully explore the efficacy of three MET inhibitors in combination with RT against MET-driven pHGG.

Detailed pharmacokinetic analyses are critical to identify the optimal MET inhibitor for brain tumor therapy. Here, we firstly describe capmatinib’s CNS penetration in mice and provide an assessment of crizotinib and capmatinib pharmacokinetic properties. For *in vivo* studies, we used a crizotinib dose previously reported to be tolerated and efficacious in mice [36], which provided a high total plasma AUC of 64,700 hr-ng/mL. Notably, the maximum tolerated dose (280 mg/m^2^ BID) for pediatric solid tumors provided a mean steady total plasma AUC of 6,990 hr-ng/mL[37]. Thus, the utilized doses in mice far exceeded clinically achievable doses, even when adjusting for the approximately 2.5-fold higher plasma protein binding of crizotinib in mice versus humans [38]. In contrast, capmatinib is administered orally at 400 mg BID in humans [15, 39], achieving a mean steady total plasma AUC of 17,300 hr-ng/mL [40] – similar to our estimated murine total plasma AUC of 16,400 hr-ng/mL. In this case, comparisons using total AUCs are appropriate, as the plasma protein binding of capmatinib is similar between mice and humans [41]. Therefore, our 25 mg/kg BID regimen of capmatinib was clinically relevant and provided plasma exposures in mice similar to patients at the approved dose.

We also compared the fractions of unbound capmatinib and crizotinib in mouse brain homogenates and found that the unbound fraction of capmatinib was 8.2-times higher than crizotinib, which likely contributes to the higher *in vivo* efficacy of capmatinib*.* Because of this higher unbound fraction, capmatinib reached a higher effective exposure in the murine brain for up to 8 hours after administration, even though crizotinib achieved much higher plasma AUCs.

The PDOX models allowed us to compare our preclinical results to the presented patient’s clinical outcome. After an initial relapse, the patient was treated with cabozantinib, based on previous clinical studies that showed activity against intracranial metastases [42, 43]. Importantly, our *in vivo* PDOX response to cabozantinib, while statistically significant, provided a brief extension of survival that would be biologically inadequate when considered as a patient outcome. Thus, our PDOX model recapitulated the clinical failure of cabozantinib, while capmatinib monotherapy induced stable disease in the PDOX model. It is possible that previous brain metastases were more responsive to cabozantinib because of a higher sensitivity to low-level MET inhibition. Alternatively, differences in the blood-brain barrier in brain metastases compared to pHGG may have allowed greater drug availability in the tumor. These examples highlight the utility of evaluating relevant models for specific diseases, even if a drug has proved efficacious in a different tumor type with a common RTK target.

We investigated the efficacy of capmatinib, crizotinib and cabozantinib *in vitro* and *in vivo* to assess disrupted signaling of downstream effectors. Although our RNAseq data demonstrated that all three drugs induced a shared cellular response at respective EC90 concentrations, capmatinib displayed a much greater potency in all examined instances, compared to crizotinib and cabozantinib. This is in agreement with previous reports that demonstrated 10 to 100-fold lower IC50 values of *in vitro* MET inhibition for capmatinib compared to crizotinib or cabozantinib [44–46], although different assays have been utilized in these studies. While it was shown that crizotinib and cabozantinib inhibit a broader spectrum of tyrosine kinases [47, 48], capmatinib has been demonstrated to selectively target MET with K_D_ values 1000-fold below its second most high-affinity target[49]. The plateau at ~20% cell viability/ abundance at higher capmatinib concentrations, which we observed in our dose response assays has been reported before [49] and is likely a result of growth arrest induced by capmatinib’s specificity, in contrast to crizotinib and cabozantinib, which also target additional tyrosine kinases at high doses and thereby induce cell death in a non-specific manner.

Dose and safety data for capmatinib treatment in children is not yet available. The FDA approved capmatinib for adults with metastatic non-small cell lung cancer (mNSCLC) with MET exon 14 skipping mutations based on a clinical trial in which capmatinib was permanently discontinued in 16% of mNSCLC patients due to an adverse reaction, most commonly pneumonitis (1.8%), peripheral edema (1.8%) and fatigue (1.5%) [50], providing initial insights into potential toxicities in the pediatric population.

Capmatinib was even more effective when administered concomitantly with radiation, which we initially demonstrated *in vitro* for all aforementioned models. In the subsequent preclinical allograft trial, capmatinib and RT increased the survival-rate and -time compared to single treatments. In the PDOX study, the combination induced full responses in all but one treated animal, whereas none of the single-treated mice displayed significant tumor regression. This outstanding efficacy of the combination in the PDOX study compared to the allograft trial may be explained in part by the different underlying treatment schedules, which were adjusted in the PDOX study based on capmatinib’s PK profile and to match a patient-equivalent dose based on a published clinical trial. Our results firstly and extensively highlight the striking advantage of combining capmatinib and RT against pHGG, and are in accordance with previous reports that demonstrated radiosensitization by MET inhibition [51–53]. Additional prior studies indicated that this effect is p53-dependent[54]. However, here we demonstrated combinatorial efficacy between capmatinib and radiation in both human *TP53*-expressing cells and in murine *Trp53*-deficient tumors, although we observed a differential expression of p53 regulating kinases after capmatinib treatment.

When analyzing the underlying mechanisms of radiosensitization we found a significant downregulation of specific DNA repair genes in capmatinib-treated tumor cells. This is in agreement with previous reports that displayed radiosensitization by downregulation of DNA repair genes after inhibition of MET [55–57] but also after inhibition of other RTKs [16, 58]. Many reports identified an involvement of ATM and ATR [17, 18, 59], which we also noted. However, the broader range of downregulated DNA repair genes together with the tight correlation between cell cycle progression and DNA repair gene expression that was observed in this study, might suggest a more general paradigm of radiosensitization by RTK-inhibition. The sudden downregulation of certain DNA repair genes, within the previously quickly proliferating tumor cells might render these cells generally more susceptible to RT. In agreement with this notion, we observed that capmatinib indeed potentiates radiation induced DNA damage in tumor cells *in vitro*. We also showed that tumors treated *in vivo* with combined capmatinib and RT contained increased levels of phosphorylated Kap1 (pS824) compared to tumors treated with vehicle and RT. This ATM-dependent phosphorylation event [29] further demonstrates the elevated DNA double-strand break signaling when combining capmatinib with RT *in vivo* and shows that effects of the combination are more than additive. This has important implications for the relative timing of drug and radiation delivery. Additional *in vivo* studies would be needed to comprehensively elucidate all aspects of the underlying signaling cascade and mechanisms driving the cooperative effects of capmatinib with irradiation. The increased efficacy of this combined therapy merits further investigation to comprehensively identify susceptible tumor entities. For example, secondary adult glioblastoma, in which MET-fusions have been identified in up to 15% [9], potentially represent another promising and eligible entity for concomitant capmatinib-radiation treatment in addition to pHGG.

Our preclinical testing in a MET-fusion IHG PDX, showed that low-dose radiation combined with capmatinib reduced tumor burden, leading to long-term progression and metastasis-free survival. To minimize radiotherapy-associated late effects, chemotherapy-based treatment approaches following surgical resection when feasible have historically been used to defer or delay RT until the age of 3-5 years or at relapse [60–63]. For children in this most vulnerable age group, capmatinib alone may provide a useful approach to reduce morbidity by delaying surgery or as a bridging therapy until an age in which combination with radiation becomes more feasible. The low-dose radiation regimen employed in our human xenograft trials and its significant potentiation with MET inhibition highlights a potentially promising approach for older pediatric and young adult populations with MET-fusion driven tumors who will be otherwise treated with only involved field radiation as a standard of care. Clinical evaluation of this regimen should be reserved for those patients old enough for consideration of radiation therapy or in those that have progressed beyond the reach of successful systemic therapy options. The optimal incorporation of capmatinib in frontline treatment for pHGG with MET fusions as neoadjuvant, adjuvant, or radiation-delaying strategy must be tested in controlled and well-monitored clinical trials.

## Conclusions

In conclusion, we generated novel, MET-fusion-driven pHGG mouse models to identify the optimal selective inhibitor for this devastating disease. Capmatinib showed greater potency and superior pharmacokinetic properties, including a greater proportion of unbound drug in the brain, when compared with crizotinib and cabozantinib. Combination of capmatinib with low-dose radiation potentiated RT-induced DNA damage and induced robust tumor regression *in vivo*, while treatment with cabozantinib recapitulated the lack of efficacy seen in the patient. Our consistent results of preclinical data using two independent and complementary mouse models provide a strong rationale for combining capmatinib and RT as novel treatment against MET-activated pHGG.

## Supplementary Information

Additional file 1. Supplementary Methods. 

Additional file 2: Supplementary Fig.1. Capmatinib potentiates RT in vitro and in vivo. a, Schematic displaying the TFG-MET fusion gene and protein structure for the construct used for *in utero* electroporation. **b, **Western blot of (phospho-)Akt in cultured, murine tumor cells after different time points of crizotinib (cri) or capmatinib (cap) treatment at the indicated concentrations. The results show a quick and temporary inhibition of Akt by both compounds. **c, **Western blot of phosphorylated and total MET, Akt and Erk, and β-Actin, in cultured murine TFG-MET tumor cells after 2 hours of DMSO control, crizotinib (cri) or capmatinib (cap) incubation at the IC50 for each drug (124nM crizotinib and 9nM capmatinib), and a positive control concentration of 1µM. **d, **Quantification of luminescence signal of western blots in panel B normalized to the respective DMSO control. Each dot represents an individual replicate. Error bars display standard error of the mean. Statistical significance was determined using a One-Way ANOVA followed by Tukey’s multiple comparisons test (*p<0.05,**p<0.01, ***p<0.001, ****p<0.0001). **e,f,** 3D illustrations of cell numbers 7 days after treatment with crizotinib and RT (D) or capmatinib and RT (E), respectively. Compound concentrations comprised 10µM and 9 serial 1:3 dilutions in 4 replicates each and radiation doses ranged from 0Gy – 8Gy. Cell numbers were determined with the CellTiter-Glo Assay. **g, **3D illustrations of ZIP synergy scores 7 days after treatment with crizotinib and RT (lower panel) or capmatinib and RT (upper panel), respectively. Tested compound concentrations comprised 10 µM and 9 serial 1:3 dilutions in 4 replicates each. ZIP synergy scores were calculated based on cell numbers according to the CellTiter-Glo Assay by using the synergyfinder tool.

Additional file 3: Supplementary Table 1. NCA PK parameter estimates for capmatinib and crizotinib in mouse plasma and brain. See Methods – Pharmacokinetics section for a description of abbreviations. Terminal phase parameters for crizotinib in brain could not be estimated and were not reported.

Additional file 4: Supplementary Table 2. EC50 and EC90 values of capmatinib (cap) and crizotinib (cri) following 3-day dose response assays. To calculate unbound EC50 and EC90 values, total values were multiplied with unbound drug fractions in culture media (Table 1).

Additional file 5: Supplementary Fig.2. Survival, body weight and tumor burden data from TFG-MET allograft-bearing mice.** a,**Schematic illustrating the preliminary in vivo study to determine the efficacy of RT alone.** b, **Survival curve of mice depicted in a. Radiation with 20 Gy lead to an increased survival time and resulted in complete tumor remission in 2 out of 5 mice. **c,** Mouse weights of mice from the Survival cohort over time. No treatment resulted in global weight loss or any grossly detectable side effects. **d,** Development of BLI signals of all enrolled mice during the course of the preclinical allograft trial. Each line represents one mouse. The ends of lines indicate the onset of neurological symptoms and thereby the endpoints. In contrast to all other treatments, capmatinib + RT induced a (temporal) remission in 8/10 mice.

Additional file 6: Supplementary Fig.3. Low magnification images of the immunohistochemical staining shown in Fig. 3d. IHC of phosphoproteins in TFG-MET tumors of the PD cohort, which were treated with the indicated therapies. Scale bar is 750 µm.

Additional file 7: Supplementary Table 3. Bioluminescence signals of *in vivo* trials.

Additional file 8: Supplementary Fig.4. Characterization of human tumor samples and derived cell cultures.**a, **H&E staining of the representative sections from the initial tumor (TRIM24-MET-i). The upper panel shows areas in the parietal region, the lower panel in the occipital region, both displaying variable histologies, compact (left) and infiltrative (right) tumor cells. Scale bar=50 µm. **b,** H&E staining of four representative sections from the recurrent tumor (TRIM24-MET-r), showing diverse cytomorphology and growth patterns. Scale bar=50µm. **c,** Initial (TRIM24-MET-i) and recurrent tumor (TRIM24-MET-r), showing punctuated GFAP expression (upper panel). Ki-67 staining indicates that most tumor cells are actively proliferating (the lower panel). Scale=50µm. **d,** Sanger sequencing results of RT-PCR amplicons, demonstrating the TRIM24-MET fusion junction in the initial (TRIM24-MET-i) and recurrent (TRIM24-MET-r) tumor samples. **e,** RT-QPCR data demonstrating the relative expression levels (normalized to GAPDH) of the TRIM24 N-terminal region, c-MET N-terminal region, MET-kinase domain and the TRIM24-MET fusion in TRIM24-MET-i and TRIM24-MET-r cells as well as in control pHGG tumor cells without TRIM24-MET fusions (SJHGGx6c, SJDIPGx37c). **f,** Immunoprecipitation (IP)-Western blots confirming the existence of TRIM24-MET protein in initial (TRIM24-MET-i) and recurrent tumor (TRIM24-MET-r) cells. Pro-MET (pM=170kD) and the mature c-MET protein (M=140kD) were identified in SJHGGx6c cells (c-MET-expressing tumor cells), and the TRIM24-MET fusion (TM=117kD) in TRIM24-MET-i and TRIM24-MET-r cells. SJDIPGx37c cells were used as a negative control of endogenous c-MET expression. **g,** IP-Western blot showing existence of TRIM24-MET. The same protein samples in “D” were blotted with a rabbit poly clonal antibody recognizing the N-terminus of TRIM24. The Western blot identifies the TRIM24-MET protein in TRIM24-MET-i and TRIM24-MET-r cells but not in control cells (SJHGGx6c and SJDIPGx37c). **h,** *In vitro* proliferation rate of initial tumor cells (TRIM24-MET-i) and recurrent tumor cells (TRIM24-MET-r), based on luminescent cell viability assay. Values were normalized to day 0.

Additional file 9: Supplementary Fig.5. Capmatinib inhibits MET downstream pathways and is effective against further MET driven pHGG models. **a,** Western blots showing the levels of phosphorylated MET kinase domain and pERK in response to crizotinib- or capmatinib-treatment after 30 min, 2 hours and 24 hours in TRIM24-MET-i and TRIM24-MET-r cells. cri=crizotinib, cap=capmatinib. **b,** Western blots showing the levels of pAKT in response to crizotinib- or capmatinib-treatment after 30min, 2 hours and 24 hours in TRIM24-MET-i and TRIM24-MET-r cells. cri=crizotinib, cap=capmatinib. **c,d,** Dose-response curves of indicated tumor cell cultures after treatment with capmatinib. Each dot or symbol represents one biological replicate of technical triplicates. Viable cells were analyzed 72 hours after compound addition using the CellTiter-Glo Assay.

Additional file 10: Supplementary Fig.6. Combining capmatinib and RT is efficacious against human tumor cells without side effects. **a,** Western blots of PDOX tumors demonstrating complete inhibition of the autophosphorylation of TRIM24-MET and decrease of pERK and pAKT levels following three doses of 25mg/kg capmatinib (2 doses on day 1, one dose on day 2), compared to vehicle (veh) treatment. **b,** Representative IHC pictures of pERK and pAKT in two pairs of tumors treated with either vehicle or capmatinib. Scale=50µm, veh=vehicle, cap=capmatinib. **c,** BLI signals of all mice enrolled in the preclinical xenograft trial comparing cabozantinib to capmatinib. Each line represents one mouse. The ends of lines indicate the onset of neurological symptoms and thereby the endpoints. Treatment started on day 13. In contrast to all other treatments, capmatinib induced tumor regression in two of eight mice and stable disease in six out of eight mice. **d, **3D chart of combinatorial *in vitro *trials, demonstrating radiation improves the efficacy of capmatinib and crizotinib in the tested concentrations. cap=capmatinib, cri=crizotinib, RT=radiation. **e,** Body weight curves of mice in the depicted 4-arm preclinical trial, starting from the first treatment. **f,** Development of total flux (radiance, p/sec/cm2/sr) derived from BLI measurements of the cranial and spine region of all vehicle-treated mice in the preclinical trial displayed in Figure 5C.

Additional file 11: Supplementary Fig.7. Molecular effects of capmatinib and radiation identified by RNAseq of murine tumors.** a,** RNAseq coverage from 6 TFG-MET allograft tumors from both the PD and survival cohorts for the TFG-MET fusion construct, notably displaying reads spanning the TFG/MET junction in a representative tumor. **b,** RNAseq alignments from a representative TFG-MET allograft tumor at the *Trp53* locus. The CRISPR-targeted cut site is indicated, at which a 1 bp insertion was observed across tumors.** c,** Schematic indicating the analyzed groups and the comparisons performed in this figure, Figure 6 and in Extended data Fig.7. The short-term capmatinib-treatment most prominently affected 4 out of 6 analyzed tumors, which were used for comparisons “A”. **d, **Bar chart indicating a homogenous distribution of reads between all analyzed samples. **e, **Expression of Mapk pathway signature genes in all samples of both cohorts. Tumors were grouped according to the indicated treatments, irrespective of RT administration. **f, **Gene set enrichment analyses of the indicated gene sets between vehicle- and capmatinib-treated tumors. Depicted pathways were significantly downregulated after capmatinib treatment.

Additional file 12: Supplementary Fig.8. Further molecular data of murine tumors and reference cohorts.** a, **Cohort-specific heatmaps of genes forming the “POSITIVE REGULATION OF CELL CYCLE G1/S PHASE” geneset (baderlab go 2019). While most analyzed genes are downregulated upon capmatinib treatment in the PD cohort, their expression is tumor-specific and highly heterogenous in the Survival cohort. **b, **Gene set enrichment analyses of the indicated gene sets between vehicle- and capmatinib-treated tumors of the PD cohort. The Atr- and Atm-pathways are downregulated after capmatinib treatment. **c, **Gene set enrichment analysis of the indicated gene set between irradiated and non- irradiated tumors of the Survival cohort. Expression of genes involved in cell cycle progression is elevated following RT. **d,** Correlation between total expression scores of the genesets “DNA REPAIR_7” and “CELL CYCLE_7” (baderlab pathways 2019) amongst all samples in the two depicted datasets.** e,** Volcano plot indicating all differentially expressed genes between capmatinib- and vehicle-treated allograft tumors. Each dot represents one gene. Red dots (“PID_P53_REGULATION_PATHWAY_3” genes (baderlab pathways 2019)) indicate that most genes involved in regulation of Tp53 signaling are downregulated following capmatinib treatment. **f,** Expression of Trp53rka and Trp53rkb in indicated tumors of the PD cohort. Both genes are downregulated after capmatinib treatment.

Additional file 13: Supplementary Table 4. Genesets from heatmap in Fig. 7b.  List of genes within the geneset “DNA REPAIR_7” as part of “baderlab pathways 2019”, which form the heatmap in Fig. 7b ordered from top to bottom.

Additional file 14: Supplementary Table 5. EC50 and EC90 values of capmatinib (cap), crizotinib (cri) and cabozantinib (cabo) following 3-day dose response assays. To calculate unbound EC50 and EC90 values, total values were multiplied with unbound drug fractions in culture media (Table 1).

Additional file 15: Supplementary Fig. 9. RNAseq of *in vitro* and *in vivo* capmatinib-treated MET fusion models identifies common transcriptomic targets. **a,** Scatter plots comparing differential gene expression analysis results from TRIM24-MET-i cells (upper panels) or TRIM24-MET-r cells (lower panels) treated with the depicted MET inhibitors *in vitro* (Cap - capmatinib; Cabo - cabozantinib; Cri – crizotinib; Veh - DMSO). Significant differentially expressed genes (adj. *p* < 0.05) for the x-axis comparison are colored orange, while those significantly differentially expressed in the y-axis comparison are colored purple. Green points are genes significantly differentially expressed in both comparisons. The number of unique and shared differentially expressed genes between each comparison are shown in parentheses. A linear regression line is depicted along with Pearson’s correlation coefficient (r) and its associated p-value. Open triangles indicate genes beyond the axis-limits. **b,** Bar plots showing significant (adj. *p* < 0.05) GSEA results for MSigDB Hallmark genesets for indicated cells and treatment comparisons (TRIM-MET cells were treated *in vitro, *TFG-MET cells *in vivo*). Common negatively enriched genesets between all three comparisons are in bold. **c,** Heatmaps showing expression of Hallmark_Myc_Target_v1/2 genes for indicated cells and treatment comparisons (TRIM-MET cells were treated *in vitro, *TFG-MET cells *in vivo*). Select leading edge genes from GSEA are labeled.

Additional file 16: Supplementary Table 6.Differential expression analysis results for TRIM24-MET-i and TRIM24-MET-r drug treatments and TFG-MET capmatinib treatment.

Additional file 17: Supplementary Table 7. Full GSEA results with select categories of MSigDB genesets for TFG-MET PD cohort treated with capmatinib.

Additional file 18: Supplementary Table 8. Full GSEA results with select categories of MSigDB genesets for TRIM24-MET-i drug treatments.

Additional file 19: Supplementary Table 9. Full GSEA results with select categories of MSigDB genesets for TRIM24-MET-r drug treatments.

Additional file 20: Supplementary Fig.10. Capmatinib treatment potentiates radiation-induced DNA damage.**a, **Heatmaps showing expression of Hallmark_DNA_Repair genes (n=146) for *in vitro* capmatinib treatments in cell lines derived from TRIM24-MET fusion tumors as compared to a DMSO vehicle control. Differentially expressed (p. adj < 0.05) genes in each comparison are labeled, along with RAD51 (bold and underlined). **b,** γH2AX-immunofluorescence staining of TFG-MET cells at different recovery timepoints following 8Gy-irradiation. Capmatinib (Cap)-treated cells display significantly higher levels of γH2AX compared to DMSO-treated (DMSO) cells. **c,** Quantification of γH2AX-foci in TFG-MET cells. The percentage of cells with ≥10 γH2AX-foci is significantly higher in capmatinib-treated cells (black bar) compared to DMSO-treated cells (white bar) at various time points following irradiation. Error bars display standard error of mean, statistical significance determined using t-test analysis, *;*p*<0.05. Scale bar is 10µm.

## Data Availability

Mouse and human tumor cell lines reported in this manuscript are available following completion of a material transfer agreement. Mouse RNAseq data and human and mouse DNA methylation array data has been deposited at Gene Expression Omnibus (GEO) GSE252459, and human RNAseq data has been deposited at the European Genome-Phenome Archive (EGA) EGAD50000000194 and will be made accessible prior to publication.

## Abbreviations

pHGG: Pediatric high-grade glioma
IHG: infant-type hemispheric glioma
PDOX: patient-derived orthotopic xenografts
Fu,b: fraction unbound in brain homogenate
RT: radiotherapy
PFS: progression-free survival
RTK: receptor tyrosine kinase
GSEA: gene set enrichment analyses
GTR: gross total resection
