# Supplementary material for: Capmatinib is an effective treatment for MET-fusion driven pediatric high-grade glioma and synergizes with radiotherapy
Source: Mol Cancer. 2024 Jun 7;23:123. doi: 10.1186/s12943-024-02027-6 (PMC11157767; doi:10.1186/s12943-024-02027-6)
Supplement: Supplementary file 1 — Additional file 1. Supplementary Methods. [file 12943_2024_2027_MOESM1_ESM.docx]

## Supplementary Materials and Methods

## Pharmacokinetics

Capmatinib (25mg/kg, MEDKOO, 205494, Lot BBC40125, purity 96.64%) in 0.5% methylcellulose / 0.5% polysorbate 80 or crizotinib (100mg/kg, MEDKOO, 202222, Lot TZC60627, purity 99.64%) in 0.1 N hydrochloric acid were administered to female CD-1 nude mice (Charles River) *via* oral gavage. Blood samples upon KEDTA were obtained at various times up to 16 hours post-dose, immediately processed to plasma, and stored at -80 °C until analysis. Following terminal bleeds, animals were perfused, brains were extracted and stored at -80°C. Brain samples were weighed and homogenized using a FastPrep-24 system (MP Biomedicals, Santa Ana, CA) for five cycles of 1 min vibration at 6.5 M/S speed, with 5 min in ice bath between each cycle to prevent over-heating. The homogenates were then stored at -80 °C until analysis. Plasma and brain homogenate were subjected to deproteinization and analyzed for drug concentrations using a qualified LC-MS/MS assay. For this, samples were protein precipitated with 100 μL of 0.3% formic acid in methanol. Aliquots of the supernatants were injected onto a Shimadzu LC-20ADXR high performance liquid chromatography system *via* a Shimadzu SIL-20AC XR autosampler. The LC separation was performed using a Phenomenex Luna C18(2) (3 μm, 50 mm x 2 mm) column at 50˚C with gradient elution at a flow rate of 0.4 mL/min. The binary mobile phase consisted of 0.3% formic acid in methanol: water (10:90, v/v) in reservoir A and 0.3% formic acid in methanol in reservoir B. The initial mobile phase composition was maintained at 10% B for 0.5 minutes and was followed by a linear increase to 100% B in 2 minutes. The column was then rinsed for 1 minute at 100% B and then equilibrated at the initial conditions for 1.5 minutes for a total run time of 5 minutes. Under these conditions, crizotinib eluded at 2.05 minutes, and capmatinib at 2.34 minutes. Both drugs were detected with tandem mass spectrometry using a SCIEX API 4000 in the positive ESI mode with the following mass were transitions monitored: crizotinib 450.10 -> 260.10, and capmatinib 413.10 -> 382.20. The intra-run precision and accuracy was ≤ 2.98% CV and 87.8% to 115%, respectively for crizotinib and ≤ 9.96% CV and 85.6% to 108%, respectively for capmatinib.

Crizotinib and capmatinib plasma and brain homogenate concentration-time (Ct) data were grouped by nominal time point, and the mean Ct values were subjected to noncompartmental analysis (NCA) using Phoenix WinNonlin 8.1 (Certara USA, Inc., Princeton, NJ). The extravascular model was applied, and area under the Ct curve (AUC) values were estimated using the “linear up log down” method. The terminal phase was defined as at least three decreasing time points at the end of the Ct profile, and the elimination rate constant (Kel) was estimated using an unweighted log-linear regression of the terminal phase. The terminal elimination half-life (T1/2) was estimated as 0.693/Kel, and the AUC from time 0 to infinity (AUCinf) was estimated as the AUC to the last time point (AUClast) + Clast (predicted)/Kel. Other parameters estimated included observed maximum concentration (Cmax), time of Cmax (Tmax), concentration at the last observed time point (Clast), time of Clast (Tlast), apparent clearance (CL/F = Dose/AUCinf), and apparent terminal volume of distribution (Vz/F). The apparent plasma-to-brain partition coefficients (Kp,inf) were estimated as the ratio of the AUCinf in brain to AUCinf plasma, whereas Kp,last was similarly estimated using AUClast values.

## Mouse model establishment

For *in utero* electroporation, the vectors pX330-Trp53, pCAGGS-T2TP and pt2k-TFG-MET-IRES-luciferase were co-delivered. PX330-Trp53, pt2k-IRES-luciferase and pCAGGS-T2TP have been described in a previous study[1]. The vector pt2k-TFG-MET-IRES-luciferase was constructed by cloning the human *TFG-MET* fusion gene with C-terminal HA tag [2] in between the EcoRI and XhoI site of the vector pt2k-IRES-luciferase. *In utero* electroporations were conducted as previously described[1] with the following exceptions. DNA was injected into the lateral ventricle. E14.5 embryos and 28V were used. At p3, successful electroporation was determined by bioluminescence imaging and only positive pups were kept. After reaching humane endpoint criteria, tumors were dissected, dissociated and cryopreserved. Cells were thawed and expanded *in vitro* before cryopreservation in multiple aliquots for preclinical studies. Two weeks prior to transplantation, cells we thawed and either passaged or treated with fresh medium twice a week. In total, cells underwent 5 *in vitro* passages before engraftment. For orthotopic transplantation into 6-8 week old WT CD1 mice, 1.5x10^5^ cells were intracranially injected at ML 1.5, AP -2.5, DV 2.5.

The TRIM24-MET-i (SJHGGx97) PDOX model was initially established by implantation of biopsy tissue into recipient CD1-nude mice at 8 weeks of age. The human tumor cells were obtained by enzymatic dissociation. Therefore, 1% Papain in Neurobasal™ media was activated with 0.16mg/ml N-acetyl cysteine for 10-15min at 37°C. The enzyme solution was sterilized by passing through a 0.22m syringe filter. The tumor tissue was digested in the enzyme solution with 0.012mg/ml of DNase for 15-30min at 37°C. Cells were passed through a 0.4 µm strainer, washed in the Neurobasal™ media, counted and resuspended in Matrigel (BD Biosciences). 0.25 x 10^6^ cells per mouse were intracranially implanted into the parietal lobe of 6- to 8-week old athymic mice (CD-1 nude, Charles River Laboratories). To expand the line, PDOX tumors were dissected from the endpoint mice, dissociated and directly re-implanted into 5-10 recipient mice. Alternatively, tumor cells were cryopreserved in either Millipore or Sigma cell freezing medium.

## Cell culture

Murine tumors from electroporated mice were mechanically dissociated using a scalpel, pipetting and a 40-μm cell strainer. Tumor cells were kept in TSM with growth factors as previously described[3] and passaged ~twice a week using TrypLE Express Enzyme (Thermo Fisher Scientific).

The tumor cell culture TRIM24-MET-i was derived from a PDOX tumor that formed after transplantation of primary tumor cells. Mouse cell contamination was removed with Miltenyi Biotec Mouse Cell Depletion Kit. The removal of residual mouse cells was confirmed by PCR amplification of a proportion of the human *H3F3A*, but not mouse *h3f3a*. Sequences of PCR products were further verified by Sanger sequencing. The primers for human *H3F3A* were *H3F3A*-F GTA AAA CGA CGG CCA GTG ATT TTG GGT AGA CGT AAT CTT CA and *H3F3A*-R CAG GAA ACA GCT ATG ACC TTT CCT GTT ATC CAT CTT TTT GTT. The mouse *h3f3a*-primers were *h3f3a*-F AGA CAC TAT CCC ACT GCT CGA CG and *h3h3a*-R GGG GCG TCT CTC TGG TTT TGG C. The TRIM24-MET-r cell line was derived directly from the surgical sample of the recurrent tumor. The cell lines were maintained and propagated in a serum-free, ingredient-defined medium that supports the growth of neural stem cells (NSC) and glial progenitor cells (GPC). The medium contained a 1:1 mixture of Neurobasal™ without phenol red (with 2% of B27 without vitamin A and 1% of N2) and ThermoFisher Knock-Out DMEM/F12 (with 2% of Stempro® neural supplement) and were supplemented with 20ng/ml of human recombinant EGF, 20ng/ml of human recombinant FGF-b, 10ng/ml of human recombinant PDGF-AA and –BB, 1% of Glutamax, 1% of sodium pyruvate, 1% of NEAA, 10mM of HEPES, 2mg/ml of heparin and 1× Primocin. Cell lines were either maintained in suspension culture as spheres or on the extracellular matrix coated (1% Geltrex) tissue culture surface. Single cells were obtained with Accutase® or Accumax®. Cells were propagated under hypoxia conditions (37°C, 5% of CO_2_ and 5% of O_2_). Cells were cryopreserved for storage in either Millipore or StemCell neural cryopreservation medium. The identity of each cell culture was examined and archived by the Promega PowerPlex Fusion® System (Promega Corporation, Madison, WI).

## Preclinical trials

For allografts, three days after transplantation, mice were imaged, ranked by signal and stratified into six groups according to an “ABCDEFFEDCBAABC…” pattern. Subsequently, treatment regimens were allocated to the six groups at random. Treatments of all groups started one week after transplantation. Mice were orally administered with either A. vehicle (0.5% Methylcellulose (type 400 cPs) / 0.5% Tween 80 in ultrapure water), B. 25mg/kg capmatinib in vehicle solution or C. 100mg/kg crizotinib in 0.1 N hydrochloric acid. Treatment continued daily with weekend pause for 12 weeks (5 days on, 2 days off). Additionally, half of the mice received fractionated radiation over a course of 6 days (5 days on, 2 days off, 1 day on) with 2 Gy per fraction and 12 Gy total. After two days of treatment, tumors of 4 representative mice per group (selected according to an “ABABABA...” pattern after BLI signal-based ranking) were preserved (procedure as described below) and referred to as “PD cohort”. These mice were sacrificed 4-6 hours after treatment. To analyze the effects of MET inhibition and radiation *in vivo* by Western blot for a quantitative assessment, the experiment was repeated with an additional 3 mice per group, which were sacrificed at 1 hour after irradiation on the second day of treatment, except for one mouse, which was sacrificed 3 hours after irradiation due to a technical issue. The remaining mice were observed until onset of neurological symptoms and constituted the “Survival cohort”. Irradiation of capmatinib-treated mice took place as early as possible (90-130 min after dosing) and irradiation of crizotinib-treated mice was performed 240-280 min post drug administration.

An additional, equally designed, preliminary trial aimed at determining the sensitivity of allografts to radiation. In this, 5 mice were treated with fractionated radiation over a course of 10 days (5 days on, 2 days off, 5 day on) with 2 Gy per fraction and 20 Gy total whereas 3 mice received no treatment as control. All mice were weighed at least once per week and monitored for 140 days after transplantation. Upon first signs of neurological symptoms, mice were anesthetized, perfused with PBS, and tumors were preserved as previously described[4].

For xenografts, TRIM24-MET-i and TRIM24-MET-r cells were transduced with a lentiviral vector (vCL20SF2-luc2a-YFP)[5] to enable *in vivo* imaging. Female CD-1 nude mice were intracranially transplanted with 0.25×10^6^ of tumor cells per mouse. Bioluminescence Imaging was performed on Mondays and Thursdays. Before randomization, we separated the mice into three groups based on their total radiance (0.3-Ax10^7^ p/s, A-7x10^7^ p/s and >7x10^7^ p/s; depending on the cohort, A varied between 1.5 and 3.). We then followed the ARRIVE guidelines and randomized each group of mice into four arms using Rand() in Excel. The four arms depended on the specific study and are respectively described in the main text. Mice whose total radiance was above 5x10^8^ or below 0.3x10^7^ were not enrolled. Capmatinib was formulated as described afore and orally administered at 25 mg/kg BID about 8 hours apart. Cabozantinib was diluted in 10 mM HCL and orally administered at 30 mg/kg QD. RT was delivered 2 hours after the first dose of capmatinib, 0.5Gy once per day, 10 Gy total. All treatments of the preclinical trial involving RT started 18 days after transplantation. The general treatment schedule was 5-days on /2-days off. However, in the trial involving radiation, RT had to be suspended for 4 days starting day 12 of treatment because of independent, exterior factors (meteorologic disturbance). Thus, RT was administered for 20 days within a 32-day timeframe, while the treatment with capmatinib continued for 43 weeks within this study. In the trial that compared *in vivo* efficacies of cabozantinib and capmatinib, all treatments started after 13 days and continued for 19 weeks, including the aforementioned treatment pause on weekends.

Image-guided fractionated radiation was administered with a Small Animal Radiation Research Platform (SARRP®, Xstrahl Inc.). The cranium was delineated by cone-beam CT and irradiated from the olfactory bulb to the base of the skull with a 18mm x 12mm beam size using the SARRP’s Motorized Variable Collimator (MVC). Radiation was delivered via opposed lateral beams.

## DNA Methylation Profiling

## DNA methylation profiles were generated using Illumina Infinium Methylation EPIC BeadChip arrays according to the manufacturer’s instructions. Raw IDAT files from the human patient tumors and cell lines were assessed for quality control and pre-processed with the sesame package[6] (v1.14.2) with the default "QCDPB" preprocessing steps. IDAT files from published human reference cohorts[7, 8] were pre-processed similarly. Low-quality samples with a detection rate <0.7 were excluded from the downstream analysis. The reference cohorts were limited to relevant tumor subgroups (n = 1438). Methylation probes residing on sex chromosomes or that were non-specific based on a published list[9-12] were removed. Raw IDAT files from the mouse model MM285k arrays were pre-processed with the sesame package (v1.14.2) with the default "TQCDPB" preprocessing steps. Single-sample Noob normalization[13] was applied to all samples to derive beta values, from which the top 15000 most variable human probes were determined after limiting each type of tumor to a maximum of 20 to better capture variation between tumor types. Probes with any missing values across samples were removed. Of these probes, those also present on the MM285 array were retained for downstream analysis (n = 405). The normalized beta values for these probes for all samples were used to calculate 1-variance weighted Pearson correlation between samples, the result of which was provided as input to the t-SNE[14] algorithm (Rtsne v0.16) with the following parameters: perplexity = 30, theta = 0.5, max_iter = 5000, is_distance = TRUE.

## ɣH2AX staining

Millicell EZ SLIDE 4-well glass chamber slides (Millipore, PEZGS0416) were coated with 1% Geltrex, and 1.5-3x10^5 TRIM24-MET or TFG-MET cells were seeded per well. Cells were treated with either 0.1% DMSO or 0.15 µM capmatinib for 24 hours before RT. Radiation was delivered by a Gulmay D330 orthovoltage irradiator, which has a 20x20 cm field size, with 300 KV, 10 mA, dose rate 36 cGy/min. Due to their different sensitivity to radiation, TRIM24-MET cells received 4 Gy while TFG-MET cells received 8 Gy. Following irradiation, chamber slides were incubated in a 5% CO_2_, normoxia incubator for designated times. Subsequently, cells were fixed with 4% PFA and treated with Phospho-Histone H2A.X (Ser139) (20E3) Rabbit mAb (CellSingaling #9718), 1:500, followed by staining with secondary antibody (Alexa Fluor® 488 (ThermoFisher A-11034) 1:800 and DAPI (ThermoFisher D21490). Chamber slides were mounted with ProLong™ Diamond Antifade Mountant (ThermoFisher P36961). The cell images were scanned by Lionheart FX Automated Microscope (Agilent).

## Dose response (DR) assays

850 cells in 70 ml media were seeded into each well of 96-well plates, coated with 1% Geltrex. Another plate was prepared with a Corning® 96-well Clear V-Bottom 2 mL Polypropylene Deep Well Plate (Corning, 3960). This plate contained 2x designated concentrations of the compound with 10 3x serial dilutions. The negative control was 0.2% DMSO and the positive control was 5 μM THZ-1. Equal volumes of cell suspensions were then added to prepared drug plates. Drug exposure was continued for 3 to 5 days. Cell viabilities were analyzed using Promega CellTiter-Glo®. Raw luminescence RLU (relative light unit) values for each compound were normalized to obtain % change in cell viability using Prism9. For normalization the respective concentration of DMSO was used. Four technical replicates within a single biological replicate were averaged together, and the results from three independent biological replicates were pooled prior to fitting. Dose-response curves were fit using the drc package[15] in R[16]. The three-parameter model (with y0, the response without drug, set to zero) was fit using the sigmoidal function LL2.4. The EC50 was constrained to be between 10-11 and 10-4 (which approximately equated to the drug concentration range tested in these experiments), and yFin, the maximum response of the dose-response curve, was constrained to be less than or equal to zero.

For additional models (#133, #135 and SJ-GBM2), 5000 cells in 80µL TSM were seeded into 96-well plates. One day later, capmatinib was added to the wells in 8 different concentrations as triplicates. DMSO was similarly diluted and used as negative controls. Drug exposure was continued for 3 days. Promega CellTiter-Glo® assay was used as readout.

For *in vitro* combination assays including radiation, the cells in 96W plates were treated with compounds as described above. Each plate received one designated dose of radiation. 4 hours after drug addition, cell cultures were irradiated using the Gulmay D330 orthovoltage irradiator in a 20x20 cm field size, 300 KV, 10 mA, dose rate 36 cGy/min. Cells were treated in single fraction doses of 0, 1, 2, 4, 6, and 8 Gy, and kept in culture for seven days. Terminal cell viability was measured by Promega CellTiter-Glo®. Synergy scores were calculated using the ZIP model within the synergyfinder tool[17].

## Immunoprecipitation (IP) and western blot

Cells were seeded at 1×10^5^ cells per cm^2^ before treatment. By the end of the treatment, the dish (6-well plate or T25 flask) was immediately cooled on ice. The cells were homogenized in cell lysis buffer with protease and phosphatase inhibitor cocktails, followed by centrifugation at 16,000g, 4°C for 30 min. The protein concentration was determined by the Pierce Micro BCA™ Protein Assay Kit. 25mg of total protein was aliquoted to individual Eppendorf tubes mixed with equal volumes of 2X loading dye (1:1 dilute of the 4X LDS loading buffer with 1M DTT). Samples were denatured at 70°C for 15min. Antibodies and dilutions used in the Western blots were P-MET Y1234/1235 (6F11) 1:500 (ABWIZ BIO, 2216S), Phospho-Akt (Ser473) (D9E) XP® Rabbit mAb 1:500 (CELL SIGNALING 4060S), Akt (pan) (C67E7) Rabbit mAb 1:1000 (CELL SIGNALING, 4691), p44/42 MAPK (Erk1/2) 1:1000 (CELL SIGNALING, 9102), Phospho-p44/42 MAPK (Erk1/2) (Thr202/Tyr204) 1:1000 (Cell Signaling, 9101), P-ERK T202/Y204 (D13.14.4E) 1:1000 (CELL SIGNALING, 4370T), Met (D1C2) XP® Rabbit mAb 1:1000 (CELL SIGNALING 8198S/T), Rad51 antibody [N1C2] 1:1000 (Genetex, GTX100469), Monoclonal Anti-β-Actin antibody, clone AC-15, 1:1000 (Sigma-Aldrich, A5441), 1:1000 anti-phospho-KAP1-ser824 (Bethyl, A300-767A) and 1:2000 anti-KAP1 (Abcam, ab10484).

For immunoprecipitation, the cell lysate from cells cultured in T75 flasks was cleared by a centrifugation 16,000 g, at 4°C for 30 min. To perform immunoprecipitation, protein A beads were washed twice with cell lysis buffer and were prepared as the 50% slurry for subsequent use. In 1.0 ml of the cell lysate (500mg of total protein), 80ul of the slurry was added at 4°C and rocked for 30 min to preclear the protein. The pre-cleared protein was collected following a centrifugation at 14,000 g, 4°C for 10 min. The anti-MET (D1C2) rabbit monoclonal antibody 1:50 (IP) or rabbit normal IgG (negative control) was added to the cell lysate 1:50. Protein-antibody binding was achieved for 2 hours at 4°C with an end-to-end shaker. 200 ml of the slurry and 1.0 ml of the protein/antibody mixture were co-incubated for 1 hour at 4°C while shaking. The protein A beads were collected by pulse centrifugation for 5 seconds, 14,000 g at 4°C. The beads were washed with 1 ml of 1X lysis buffer with the protease inhibitor cocktail on ice, followed by pulse centrifugation for 3 more times. Samples were diluted 1:1 with 4X LDS loading buffer containing 1 M DTT to achieve a 2X reducing loading buffer. The washed beads were resuspended in 70 ml of 2X loading buffer. The protein was denatured at 95°C for 5 min. The denatured protein was collected by centrifugation 14,000 g for 5 min. The protein was aliquoted for 3 loadings. The mouse anti-MET C-terminus monoclonal antibody (L41G3 mouse mAb, 1:1000) and the rabbit anti-TRIM24 N-terminus (1:1000) were used, respectively. To avoid the signal of the rabbit IgG heavy chain on the blot, the anti-rabbit TrueBlot HRP (1:1000) was used to detect the N-TRIM24.

The protein was separated by the NuPAGE 4-12% Bis-Tris gel in 1XMOPS buffer and was blotted on PVDF membranes. Western blots were imaged with Li-Cor Odyssey® Fc.

## Immunohistochemistry

Tumors induced by *in utero* electroporation were processed, sectioned and haematoxylin-eosin (HE) stained as previously described[1]. For further immunohistochemical analyzes, antigen retrieval was performed by boiling for 20 min in citrate buffer (0.01M citric acid, 0.01M sodium citrate). After blocking, utilized primary antibodies comprised anti-pERK (Cell Signaling, 4370S for the tumors generated by IUE or Cell Signaling, 9101 for the allograft and PDOX tumors, rabbit, dilution 1:1000), anti-pAkt (Cell Signaling, 4060S, rabbit, dilution 1:200), anti-pMET (Cell Signaling, 3077S rabbit, dilution 1:320) and anti-HA (Cell Signaling, 3724S, rabbit, dilution 1:800). The secondary antibody (Donkey Anti-Rabbit, Jackson Immuno Research, 711-065-152) was diluted 1:400. Staining was visualized using the avidin-biotin peroxidase system (VectorLab, PK-6100) and freshly prepared diaminobenzidine (Nichirei Bioscience, 425312F). Slides were counterstained with haematoxylin, dehydrated and mounted.

Immunohistochemistry of patient samples was performed on 5 µm formalin-fixed and paraffin-embedded (FFPE) tissue sections to detect GFAP (clone 6F2, DAKO, M0761, 1:400) and Ki67 (clone MIB-1, DAKO, M7240, 1:200).

## ADME (adsorption, distribution, metabolism, and excretion) profiling

The protein binding assay determines the fractions of protein-bound and free drug in media, plasma, or brain homogenate using rapid equilibrium dialysis, which only allows the aqueous component of a biological matrix containing the free compound through a dialysis membrane (MWCO 8k media and plasma; MWCO 12k brain homogenate).

Compounds stocks were prepared at 10 mM in DMSO. Dulbecco's phosphate buffered saline (DPBS; pH 7.4) was obtained from Invitrogen (Carlsbad, CA). A single-Use RED (rapid equilibrium dialysis) device was obtained from Thermo scientific (Rockford, IL). Mouse plasma was purchased from GeneTex (Irvine, CA) and mouse brain homogenate was from BioIVT (Hicksville, NY). Sample preparation for protein binding was modified from Waters *et al.*[19]. Teflon-based plates with RED inserts (MWCO 8 K or 12K) were used without pre-conditioning. Each compound was prepared at 10 µM in media, plasma, or 1uM in brain homogenate (brain:PBS 1:9 w/w). This was done by adding 1 µL of drug stock (10 mM in DMSO) to 1000 µL of media or plasma (0.1% DMSO), or 1 µL of drug stock (1 mM in DMSO) to 1000 µL of brain homogenate (0.1% DMSO), respectively. Spiked solutions were placed into the sample chamber (indicated by the red ring), and adjacent chambers were filled with DPBS. The plate was sealed and incubated at 37°C on an orbital shaker (100 rpm) for 4 hours (media and plasma) or 6 hours (brain homogenate). After incubation, aliquots (50 µL) were aspirated from each side of the sample chamber and dispensed into a 96-weyll deep plate. An equal volume of blank matrix (media, plasma, or brain homogenate) or DPBS was added to the required wells to create analytically identical sample matrices (matrix matching). To each sample, 300 µL of acetonitrile was added containing 40 ng/ml warfarin as internal standard. Plates were sealed, mixed at 600 rpm for 10 min and centrifuged at 4000 rpm for 20 min. The supernatants (50 μL) were transferred to analytical plates, mixed with 50 µl MilliQ water, and then analyzed using the LC-MS/MS system. Compound concentrations were quantified in both buffer and biological matrix chambers via peak areas relative to the internal standard. The percentage of the compound bound to media, plasma or brain homogenate, respectively, was calculated by the following equation: fu,p = Concentration buffer chamber/ concentration media chamber. The free brain fraction was calculated by the following equation: fu,b = (1/D)/(((1/ fu,d)-1)+1/D), where D is dilute fraction. LC-MS/MS analysis was performed using an Acquity UPLC /6500 triple-quadrupole mass spectrometer (SCIEX, Forster City, CA). The UPLC column was maintained at 55 °C. Chromatographic separation was performed on an Acquity BEH C18 1.7 μm column (2.1 x 50 mm) (Waters Corporation, Milford, MA) by gradient elution at a constant flow rate of 0.9 mL/min for 2 min. The mobile phase consisted of 0.1% formic acid–water (solvent A) and 0.1% formic acid–acetonitrile (solvent B). The gradient applied was 0-0.2 min, B% 1-1%; 0.2-0.5min, B% 1-50%; 0.5-1.6 min, B% 50-95%; 1.6-1.95 min, B% 95-95%; 1.95-1.96 min, B% 95-1%; and 1.96-2.2 min, B% 1-1%. The first 0.4 min of eluate was desalted to waste by an integrated valve. The remaining eluates were directed to the triple quadrupole mass spectrometer, which was equipped with an electrospray ionization source. LC-MS/MS was performed in positive polarity (at 3000 V), and the source temperature was 500 °C. Gas 1 and gas 2 settings for nitrogen were set to 60. The curtain gas and collision gas were also nitrogen and were set to 30 and low, respectively. Multiple reaction monitoring transitions and energy parameters for each tested compound and controls were selected by Discovery Quant Software and reported in CDD. Data acquisition was conducted with Analyst 1.7.3 (SCIEX) and data processing was operated with MS-OS 2.1.1 software (SCIEX).

## Calculation of preclinically-relevant compound concentrations

The unbound drug-respective Cmax values (Cmax-unbound) in the mouse brain were calculated by Cmax * the unbound fraction in the brain. These equaled 1.005906648 μM * 0.0106 = 0.0107μM for crizotinib and 1.178410358 μM * 0.0872 = 0.103 μM for capmatinib. The unbound fractions (Fu) in culture media were 0.6165 for crizotinib and 0.7049 for capmatinib. To achieve the equivalent unbound drug concentrations in media, Cmax-unbound was divided by the fu. These equaled 0.0107μM /0.6165=0.0174 μM (~ 0.02 μM) of crizotinib and 0.103 μM/0.7049=0.146 μM (~ 0.15 μM) of capmatinib.

## Animal studies general aspects

All animal studies were conducted according to the guidelines and regulation of the National Institute of Health. All procedures in this study haven been approved by the Institutional Animal Care and Use Committee (IACUC) of SJCRH. De-identified specimens were used to generate patient-derived xenografts. CD-1® IGS mice and CD-1® Nude (Crl:CD1-Foxn1^nu^) mice were purchased from Charles River maintained in an accredited facility of the Association for Assessment of Laboratory Animal Care in accordance with NIH guidelines. The husbandry conditions were maintained by the Animal Resource Center as follows: temperature range 68 -74°F, humidity range 30-70%, light cycle (hours light/hours dark) 12/12, and time control (light on/off) 6 AM/6 PM. The tumor bearing mice were monitored daily for neurological symptoms and health issues. All mice were euthanized at humane endpoints (i.e., inability to obtain food or water, piloerection of fur and lack of activity, impaired mobility, loss of body weight > 20%, ataxia, spinning, hind limb paralysis, and seizures).

## Intracranial transplantation

Mice were anesthetized by intraperitoneal (IP) injection of a ketamine-xylazine cocktail (100mg/kg and 10 mg/kg, respectively) to reach a surgical plane of anesthesia as determined by loss of the pedal reflex. Sterile lubricant was applied to each eye and the scalp prepared for aseptic surgery with alternating sterile alcohol then betadine solution. Each mouse was then placed on a stereotactic frame base with a heated pad. A sagittal incision was made from the base of the skull to the posterior tip of the nasal bone and the fascia cleared by gentle application of sterile cotton swab. A rectangular window (2mm x 2mm) was cut using a dental drill (XL-30W, Osada Inc., Los Angeles, CA) with sterile burr and dura left intact prior to injection. Tumor cells suspended in Matrigel (BD Biosciences) were injected using sterile Hamilton syringe with 30-gauge needle at the depth 2-3mm using the dura touchpoint as a "zero". After injection, the incision was closed with sterile wound clips and the animal placed in cage on heating pad and monitored until ambulatory. Ibuprofen (2 ml children's Motrin (20 mg/ml) per 350ml water) was given for 48 hours. Mice were monitored for 7 days and wound clips were removed 7-10 days after surgery.

## Bioluminescence imaging

Mice were anesthetized with isoflurane (2-3% in 100% oxygen) and bioluminescence images were acquired with an IVIS Spectrum (Perkin Elmer, Waltham, MA) 10 minutes after intraperitoneal injection of Potassium D-Luciferin solution (Perkin Elmer, 3 mg/mouse). Luminescence values for all allo- and xenografts were quantified as the total radiance (photons/s/cm^2^/sr) from identical regions of interest (ROI) encompassing the mouse head using Living Image 4.7 (Perkin Elmer). For visual presentation of bioluminescent changes over time and between treatment groups, all images were normalized to the same color scale by setting maximum signal of luminescent activity as indicated in the figures and main text.

## PCR and QPCR

RNA was extracted with the Qiagen RNease plus kit. The c-DNA was generated using the SuperScript™ III First-Strand Synthesis System. QPCRs were done with Bio-Rad SYBR® Green Master Mix with the CFX96 Touch Deep Well Real-Time PCR System using the build-in standard program. Regular PCRs were done with the Phusion High-Fidelity PCR Master Mix. The PCR parameters followed the manufacturer’s instructions. The PCR product was either extracted from a 1% agrose gel or purified by Qiagen PCR QIAquick PCR Purification Kit for Sanger sequencing.

## RNA sequencing

RNA from frozen murine tumors was isolated using the AllPrep DNA/RNA/Protein Mini Kit (Qiagen) with an additional phenol–chloroform extraction after cell lysis. Libraries were prepared using the TruSeq Stranded mRNA Library Prep Kit (Illumina) and sequenced on a NovaSeq 6000 (Illumina). Data was processed, normalized and analyzed *via* the R2: Genomics Analysis and Visualization Platform (<http://r2.amc.nl>). Gene set enrichment analysis was performed as previously described[20, 21].

For human tumors, 1.5 million cells per well of TRIM24-MET-I and TRIM24-MET-r cells, were treated with either 0.1% DMSO or MET inhibitors at the respective EC90 concentration for 4 hours in triplicates. Total RNA was extracted with Qiagen RNeasy plus mini kit. Libraries were prepared from total RNA with the TruSeq Stranded Total RNA Library Prep Kit according to the manufacturer’s instructions (Illumina, PN 20020599). Paired end 100 cycle sequencing was performed on a NovaSeq 6000 (Illumina). Transcript level count estimates were generated with the nf-core RNA-seq pipeline14[22] (v3.1) using selective alignment via salmon15[23] (v1.4.0) with an hg19 decoy-aware transcriptome and Gencode v19 annotations with the --seqBias and --gcBias parameters. Transcript counts were collapsed to the gene level with the tximport[24] (v1.22.0) R package. For comparisons with human tumors, reads from TGF-MET allografts were aligned to mm10 with STAR (v2.5.3a) using Gencode M12 annotations and two pass alignment. Gene counts were generated with featureCounts[25] from the Subread package (v1.6.5) using both reads of a paired fragment and unique alignments. Differential expression analyses were performed with DESeq2[26] (v1.34.0) using an adjusted p-value threshold of 0.05. Gene set enrichment analyses were performed with fgsea[27] (v1.20.0) and the MSigDB[28] (v7.3) genesets using an adjusted p-value of 0.05 as a significance threshold. Visualizations were created with dittoSeq[29] (v1.6.0) and ggplot2[30] (v3.3.5) using either log2 normalized counts or variance stabilization transformed counts as appropriate.

## Sanger sequencing

Sanger sequencing of TRIM24-MET PCR fragments was done with the primers previously described[31]. Sanger sequencing of the successfully edited *Trp53* locus in tumors induced by *in utero* electroporation was performed as described elsewhere[1].

## Statistical analyses

Survival was defined from tumor implantation until death or end of follow-up, whichever occurred first. Mice that were alive at end of follow-up (92 days for allograft trial, 338 days for xenograft trial that included cabozantinib) were treated as censored observations. Kaplan Meier approach was used to plot the data and log rank tests were used for survival comparisons. P-values< 0.05 were considered significant.

## References

1. Zuckermann M, Hovestadt V, Knobbe-Thomsen CB, Zapatka M, Northcott PA, Schramm K, Belic J, Jones DTW, Tschida B, Moriarity B, et al: **Somatic CRISPR/Cas9-mediated tumour suppressor disruption enables versatile brain tumour modelling.** *Nature Communications* 2015, **6:**7391.

2. International Cancer Genome Consortium PedBrain Tumor P: **Recurrent MET fusion genes represent a drug target in pediatric glioblastoma.** *Nat Med* 2016, **22:**1314-1320.

3. Lin GL, Monje M: **A Protocol for Rapid Post-mortem Cell Culture of Diffuse Intrinsic Pontine Glioma (DIPG).** *Journal of visualized experiments : JoVE* 2017**:**55360.

4. Larson JD, Kasper LH, Paugh BS, Jin H, Wu G, Kwon C-H, Fan Y, Shaw TI, Silveira AB, Qu C, et al: **Histone H3.3 K27M Accelerates Spontaneous Brainstem Glioma and Drives Restricted Changes in Bivalent Gene Expression.** *Cancer cell* 2019, **35:**140-155.e147.

5. Alexander TB, Gu Z, Iacobucci I, Dickerson K, Choi JK, Xu B, Payne-Turner D, Yoshihara H, Loh ML, Horan J, et al: **The genetic basis and cell of origin of mixed phenotype acute leukaemia.** *Nature* 2018, **562:**373-379.

6. Zhou W, Triche TJ, Jr., Laird PW, Shen H: **SeSAMe: reducing artifactual detection of DNA methylation by Infinium BeadChips in genomic deletions.** *Nucleic Acids Res* 2018, **46:**e123.

7. Capper D, Jones DTW, Sill M, Hovestadt V, Schrimpf D, Sturm D, Koelsche C, Sahm F, Chavez L, Reuss DE, et al: **DNA methylation-based classification of central nervous system tumours.** *Nature* 2018, **555:**469-474.

8. Clarke M, Mackay A, Ismer B, Pickles JC, Tatevossian RG, Newman S, Bale TA, Stoler I, Izquierdo E, Temelso S, et al: **Infant High-Grade Gliomas Comprise Multiple Subgroups Characterized by Novel Targetable Gene Fusions and Favorable Outcomes.** *Cancer Discov* 2020, **10:**942-963.

9. Benton MC, Johnstone A, Eccles D, Harmon B, Hayes MT, Lea RA, Griffiths L, Hoffman EP, Stubbs RS, Macartney-Coxson D: **An analysis of DNA methylation in human adipose tissue reveals differential modification of obesity genes before and after gastric bypass and weight loss.** *Genome Biol* 2015, **16:**8.

10. Chen YA, Lemire M, Choufani S, Butcher DT, Grafodatskaya D, Zanke BW, Gallinger S, Hudson TJ, Weksberg R: **Discovery of cross-reactive probes and polymorphic CpGs in the Illumina Infinium HumanMethylation450 microarray.** *Epigenetics* 2013, **8:**203-209.

11. Pidsley R, Zotenko E, Peters TJ, Lawrence MG, Risbridger GP, Molloy P, Van Djik S, Muhlhausler B, Stirzaker C, Clark SJ: **Critical evaluation of the Illumina MethylationEPIC BeadChip microarray for whole-genome DNA methylation profiling.** *Genome Biology* 2016, **17:**208.

12. McCartney DL, Walker RM, Morris SW, McIntosh AM, Porteous DJ, Evans KL: **Identification of polymorphic and off-target probe binding sites on the Illumina Infinium MethylationEPIC BeadChip.** *Genomics Data* 2016, **9:**22-24.

13. Triche TJ, Jr., Weisenberger DJ, Van Den Berg D, Laird PW, Siegmund KD: **Low-level processing of Illumina Infinium DNA Methylation BeadArrays.** *Nucleic Acids Res* 2013, **41:**e90.

14. Maaten LVD: **Accelerating t-SNE using tree-based algorithms.** *J Mach Learn Res* 2014, **15:**3221–3245.

15. Ritz C, Streibig JC: **Bioassay Analysis Using R.** *Journal of Statistical Software; Vol 1, Issue 5 (2005)* 2005.

16. Team R: **A language and environment for statistical computing.** *Computing* 2006, **1**.

17. Ianevski A, Giri AK, Aittokallio T: **SynergyFinder 2.0: visual analytics of multi-drug combination synergies.** *Nucleic Acids Research* 2020, **48:**W488-W493.

18. Schindelin J, Arganda-Carreras I, Frise E, Kaynig V, Longair M, Pietzsch T, Preibisch S, Rueden C, Saalfeld S, Schmid B, et al: **Fiji: an open-source platform for biological-image analysis.** *Nature Methods* 2012, **9:**676-682.

19. Waters NJ, Jones R, Williams G, Sohal B: **Validation of a rapid equilibrium dialysis approach for the measurement of plasma protein binding.** *J Pharm Sci* 2008, **97:**4586-4595.

20. Subramanian A, Tamayo P, Mootha VK, Mukherjee S, Ebert BL, Gillette MA, Paulovich A, Pomeroy SL, Golub TR, Lander ES, Mesirov JP: **Gene set enrichment analysis: A knowledge-based approach for interpreting genome-wide expression profiles.** *Proceedings of the National Academy of Sciences* 2005, **102:**15545.

21. Mootha VK, Lindgren CM, Eriksson K-F, Subramanian A, Sihag S, Lehar J, Puigserver P, Carlsson E, Ridderstråle M, Laurila E, et al: **PGC-1α-responsive genes involved in oxidative phosphorylation are coordinately downregulated in human diabetes.** *Nature Genetics* 2003, **34:**267-273.

22. Ewels PA, Peltzer A, Fillinger S, Patel H, Alneberg J, Wilm A, Garcia MU, Di Tommaso P, Nahnsen S: **The nf-core framework for community-curated bioinformatics pipelines.** *Nature Biotechnology* 2020, **38:**276-278.

23. Patro R, Duggal G, Love MI, Irizarry RA, Kingsford C: **Salmon provides fast and bias-aware quantification of transcript expression.** *Nature methods* 2017, **14:**417-419.

24. Soneson C, Love MI, Robinson MD: **Differential analyses for RNA-seq: transcript-level estimates improve gene-level inferences.** *F1000Res* 2015, **4:**1521.

25. Liao Y, Smyth GK, Shi W: **featureCounts: an efficient general purpose program for assigning sequence reads to genomic features.** *Bioinformatics* 2014, **30:**923-930.

26. Love MI, Huber W, Anders S: **Moderated estimation of fold change and dispersion for RNA-seq data with DESeq2.** *Genome Biology* 2014, **15:**550.

27. Korotkevich G, Sukhov V, Budin N, Shpak B, Artyomov MN, Sergushichev A: **Fast gene set enrichment analysis.** *bioRxiv* 2021**:**060012.

28. Liberzon A, Birger C, Thorvaldsdóttir H, Ghandi M, Mesirov JP, Tamayo P: **The Molecular Signatures Database (MSigDB) hallmark gene set collection.** *Cell Syst* 2015, **1:**417-425.

29. Bunis DG, Andrews J, Fragiadakis GK, Burt TD, Sirota M: **dittoSeq: universal user-friendly single-cell and bulk RNA sequencing visualization toolkit.** *Bioinformatics* 2020, **36:**5535-5536.

30. Wickham H: *ggplot2: Elegant Graphics for Data Analysis.* Springer International Publishing; 2016.

31. Hiemenz MC, Skrypek MM, Cotter JA, Biegel JA: **Novel TRIM24-MET Fusion in a Neonatal Brain Tumor.** *JCO Precision Oncology* 2019**:**1-6.
