# Supplementary material for: Capmatinib is an effective treatment for MET-fusion driven pediatric high-grade glioma and synergizes with radiotherapy
Source: Mol Cancer. 2024 Jun 7;23:123. doi: 10.1186/s12943-024-02027-6 (PMC11157767; doi:10.1186/s12943-024-02027-6)

Supplementary Fig. 2

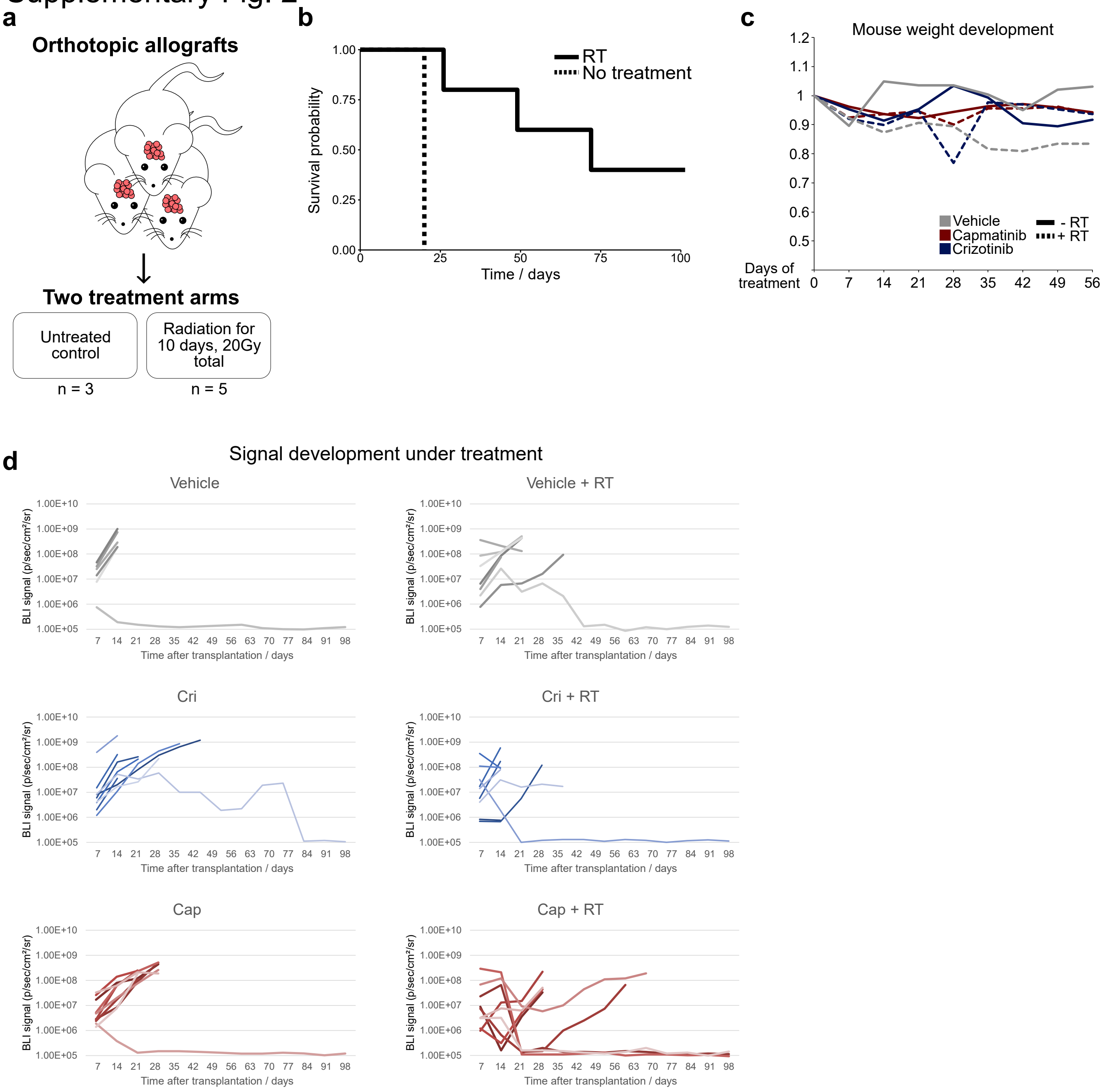

Supplement: Supplementary file 5 — Additional file 5: Supplementary Fig.2. Survival, body weight and tumor burden data from TFG-MET allograft-bearing mice. a,Schematic illustrating the preliminary in vivo study to determine the efficacy of RT alone. b, Survival curve of mice depicted in a. Radiation with 20 Gy lead to an increased survival time and resulted in complete tumor remission in 2 out of 5 mice. c, Mouse weights of mice from the Survival cohort over time. No treatment resulted in global weight loss or any grossly detectable side effects. d, Development of BLI signals of all enrolled mice during the course of the preclinical allograft trial. Each line represents one mouse. The ends of lines indicate the onset of neurological symptoms and thereby the endpoints. In contrast to all other treatments, capmatinib + RT induced a (temporal) remission in 8/10 mice. [file 12943_2024_2027_MOESM5_ESM.pdf]
