# Supplementary figures and images for: Capmatinib is an effective treatment for MET-fusion driven pediatric high-grade glioma and synergizes with radiotherapy
Source: Mol Cancer. 2024 Jun 7;23:123. doi: 10.1186/s12943-024-02027-6 (PMC11157767; doi:10.1186/s12943-024-02027-6)

**Supplementary Fig. 3**

**a**

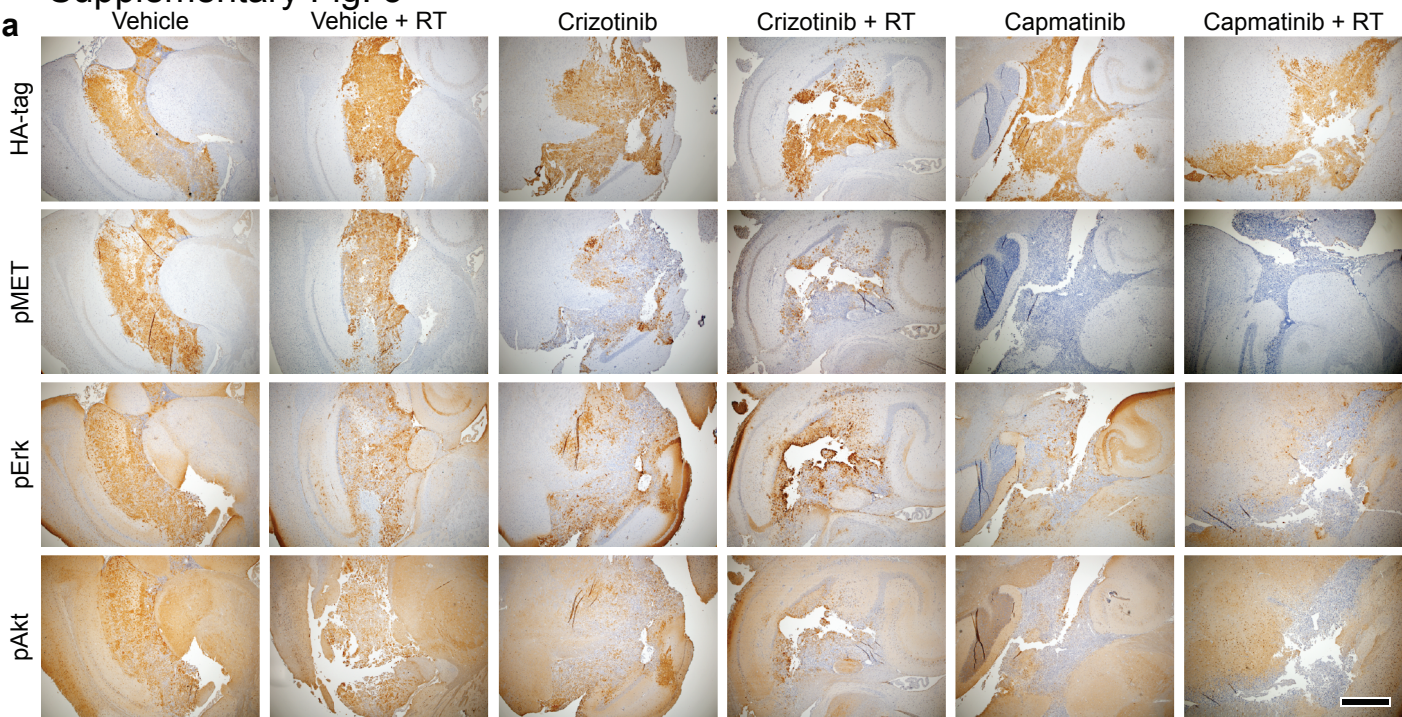

Supplement: Supplementary file 6 — Additional file 6: Supplementary Fig.3. Low magnification images of the immunohistochemical staining shown in Fig. 3d. IHC of phosphoproteins in TFG-MET tumors of the PD cohort, which were treated with the indicated therapies. Scale bar is 750 µm. [file 12943_2024_2027_MOESM6_ESM.pdf]

**Supplementary Fig. 4**

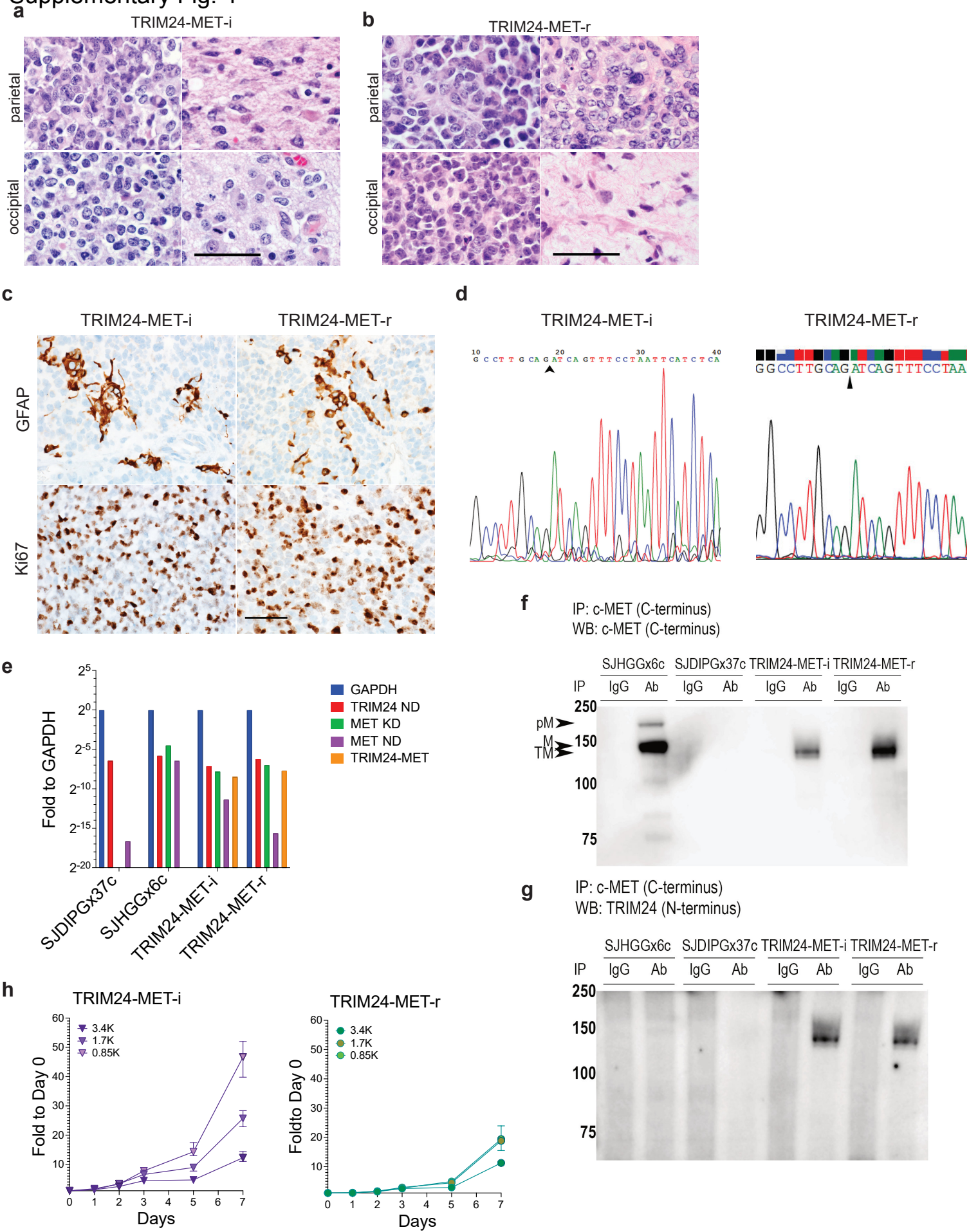

Supplement: Supplementary file 8 — Additional file 8: Supplementary Fig.4. Characterization of human tumor samples and derived cell cultures.a, H&E staining of the representative sections from the initial tumor (TRIM24-MET-i). The upper panel shows areas in the parietal region, the lower panel in the occipital region, both displaying variable histologies, compact (left) and infiltrative (right) tumor cells. Scale bar=50 µm. b, H&E staining of four representative sections from the recurrent tumor (TRIM24-MET-r), showing diverse cytomorphology and growth patterns. Scale bar=50µm. c, Initial (TRIM24-MET-i) and recurrent tumor (TRIM24-MET-r), showing punctuated GFAP expression (upper panel). Ki-67 staining indicates that most tumor cells are actively proliferating (the lower panel). Scale=50µm. d, Sanger sequencing results of RT-PCR amplicons, demonstrating the TRIM24-MET fusion junction in the initial (TRIM24-MET-i) and recurrent (TRIM24-MET-r) tumor samples. e, RT-QPCR data demonstrating the relative expression levels (normalized to GAPDH) of the TRIM24 N-terminal region, c-MET N-terminal region, MET-kinase domain and the TRIM24-MET fusion in TRIM24-MET-i and TRIM24-MET-r cells as well as in control pHGG tumor cells without TRIM24-MET fusions (SJHGGx6c, SJDIPGx37c). f, Immunoprecipitation (IP)-Western blots confirming the existence of TRIM24-MET protein in initial (TRIM24-MET-i) and recurrent tumor (TRIM24-MET-r) cells. Pro-MET (pM=170kD) and the mature c-MET protein (M=140kD) were identified in SJHGGx6c cells (c-MET-expressing tumor cells), and the TRIM24-MET fusion (TM=117kD) in TRIM24-MET-i and TRIM24-MET-r cells. SJDIPGx37c cells were used as a negative control of endogenous c-MET expression. g, IP-Western blot showing existence of TRIM24-MET. The same protein samples in “D” were blotted with a rabbit poly clonal antibody recognizing the N-terminus of TRIM24. The Western blot identifies the TRIM24-MET protein in TRIM24-MET-i and TRIM24-MET-r cells but not in control cells (SJHGGx6c and SJDIPGx37c). [file 12943_2024_2027_MOESM8_ESM.pdf]

# Supplementary Fig. 5

**a**

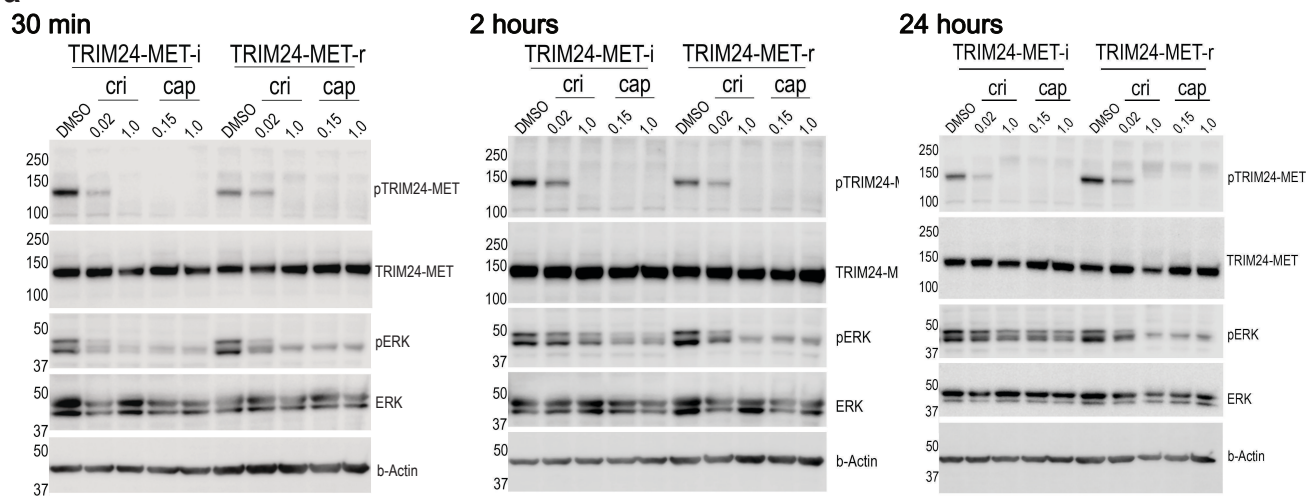

**b**

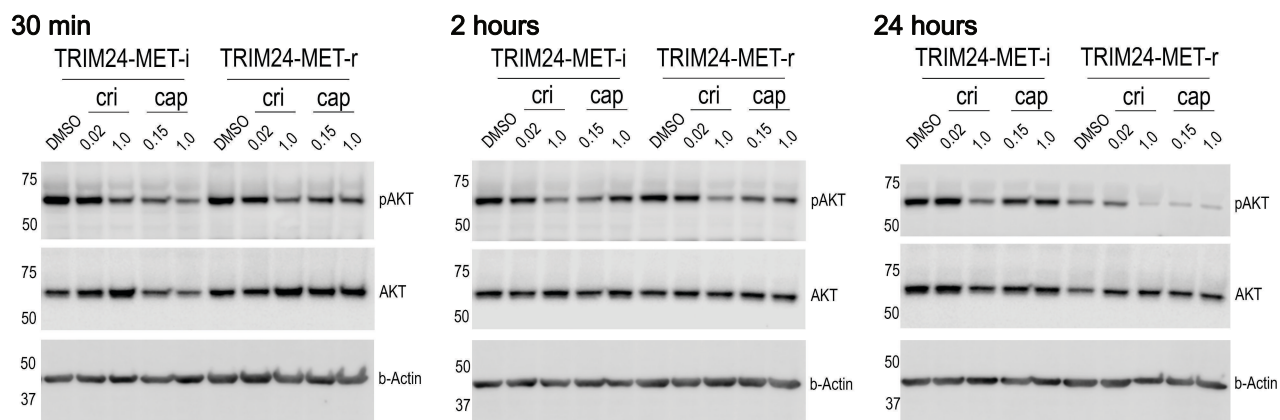

**c**

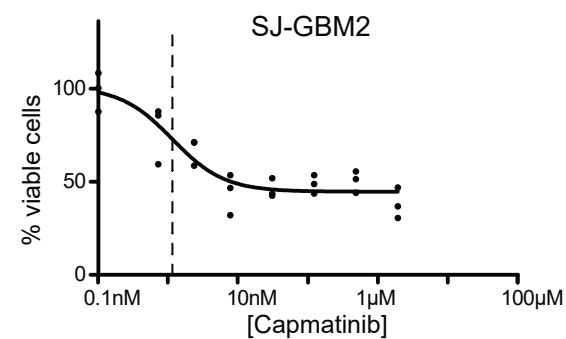

**d**

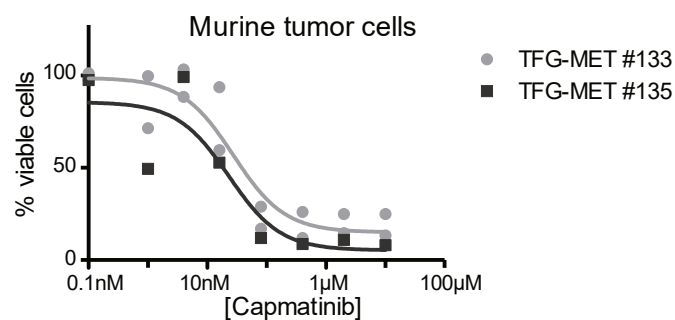

Supplement: Supplementary file 9 — Additional file 9: Supplementary Fig.5. Capmatinib inhibits MET downstream pathways and is effective against further MET driven pHGG models. a, Western blots showing the levels of phosphorylated MET kinase domain and pERK in response to crizotinib- or capmatinib-treatment after 30 min, 2 hours and 24 hours in TRIM24-MET-i and TRIM24-MET-r cells. cri=crizotinib, cap=capmatinib. b, Western blots showing the levels of pAKT in response to crizotinib- or capmatinib-treatment after 30min, 2 hours and 24 hours in TRIM24-MET-i and TRIM24-MET-r cells. cri=crizotinib, cap=capmatinib. c,d, Dose-response curves of indicated tumor cell cultures after treatment with capmatinib. Each dot or symbol represents one biological replicate of technical triplicates. Viable cells were analyzed 72 hours after compound addition using the CellTiter-Glo Assay. [file 12943_2024_2027_MOESM9_ESM.pdf]

**Supplementary Fig. 6**

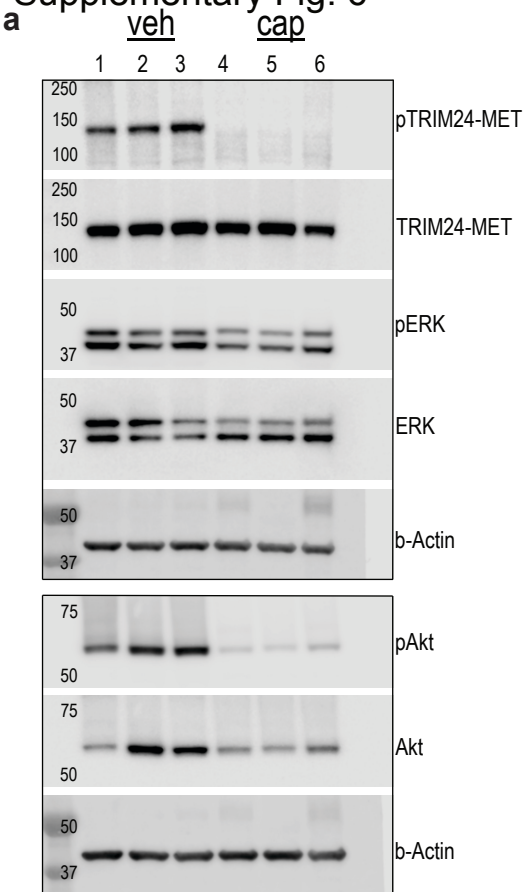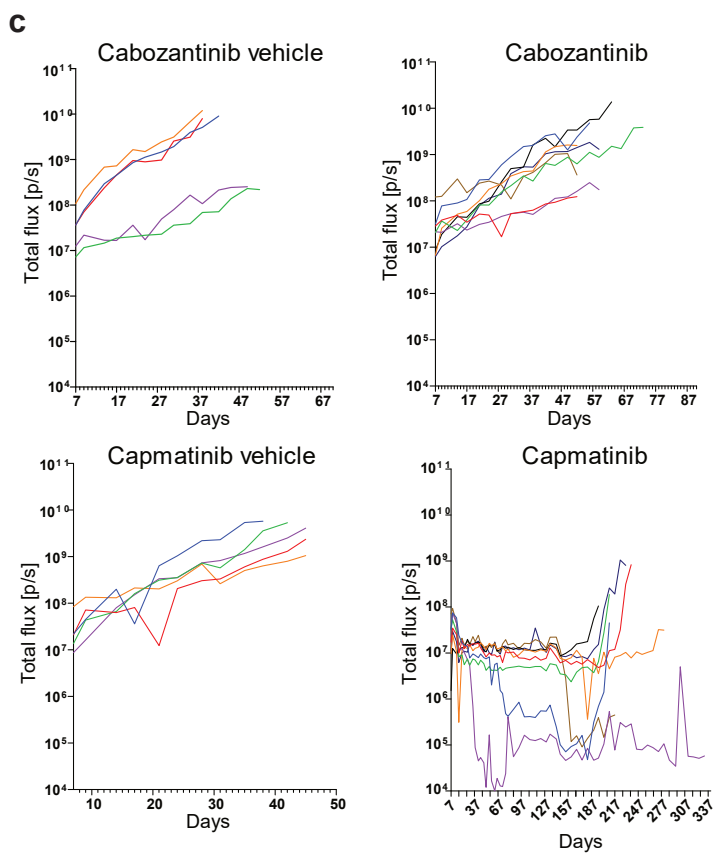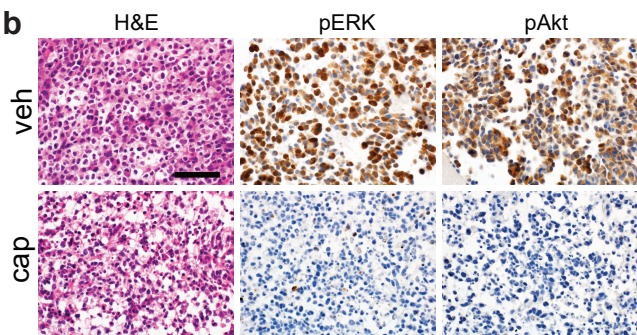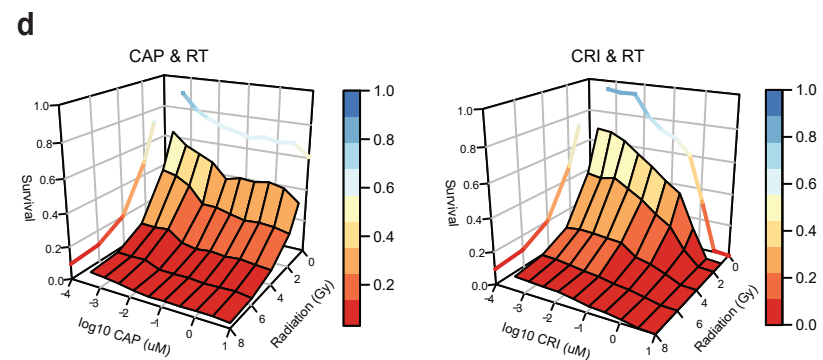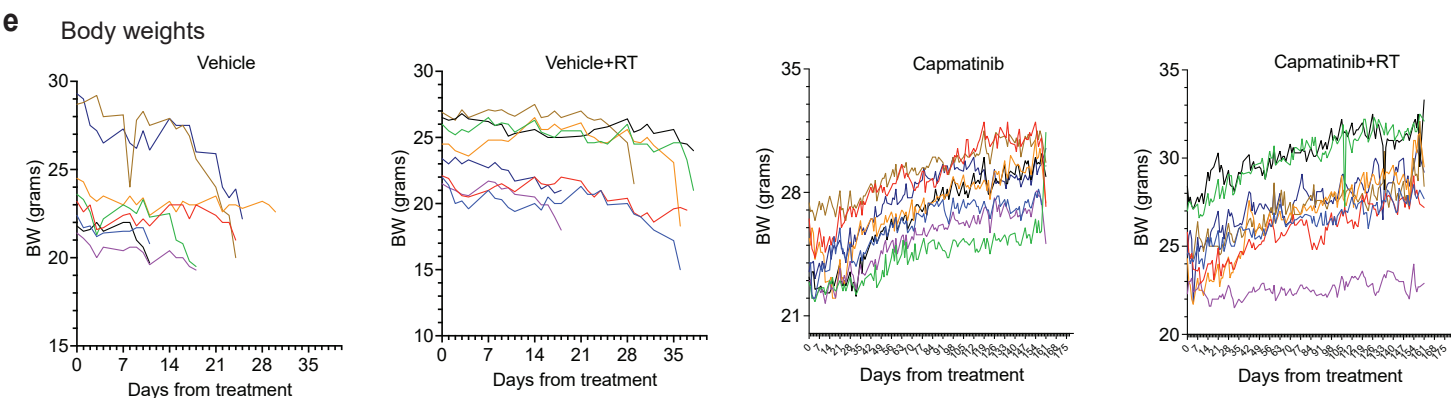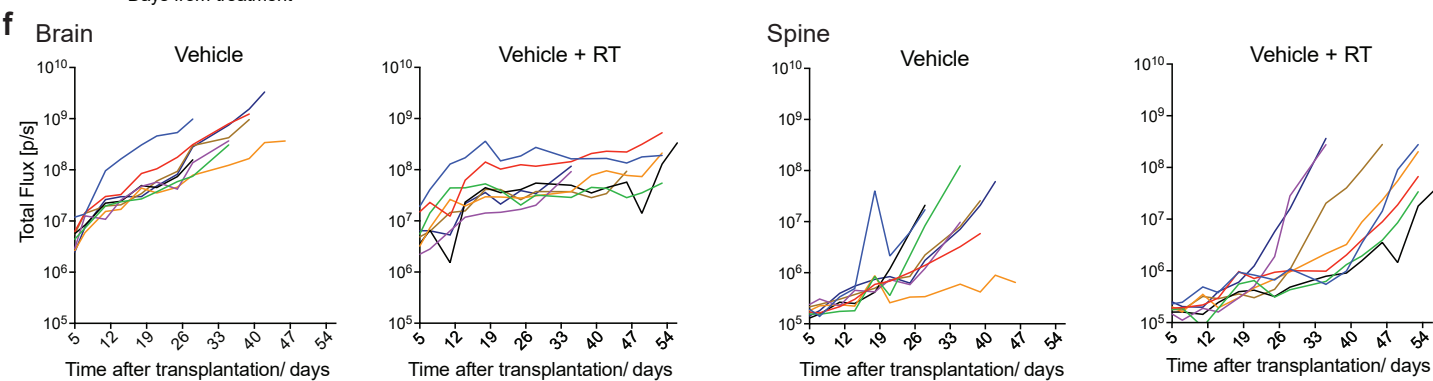

Supplement: Supplementary file 10 — Additional file 10: Supplementary Fig.6. Combining capmatinib and RT is efficacious against human tumor cells without side effects. a, Western blots of PDOX tumors demonstrating complete inhibition of the autophosphorylation of TRIM24-MET and decrease of pERK and pAKT levels following three doses of 25mg/kg capmatinib (2 doses on day 1, one dose on day 2), compared to vehicle (veh) treatment. b, Representative IHC pictures of pERK and pAKT in two pairs of tumors treated with either vehicle or capmatinib. Scale=50µm, veh=vehicle, cap=capmatinib. c, BLI signals of all mice enrolled in the preclinical xenograft trial comparing cabozantinib to capmatinib. Each line represents one mouse. The ends of lines indicate the onset of neurological symptoms and thereby the endpoints. Treatment started on day 13. In contrast to all other treatments, capmatinib induced tumor regression in two of eight mice and stable disease in six out of eight mice. d, 3D chart of combinatorial in vitro trials, demonstrating radiation improves the efficacy of capmatinib and crizotinib in the tested concentrations. cap=capmatinib, cri=crizotinib, RT=radiation. e, Body weight curves of mice in the depicted 4-arm preclinical trial, starting from the first treatment. f, Development of total flux (radiance, p/sec/cm2/sr) derived from BLI measurements of the cranial and spine region of all vehicle-treated mice in the preclinical trial displayed in Figure 5C. [file 12943_2024_2027_MOESM10_ESM.pdf]

# Supplementary Fig. 7

**a**

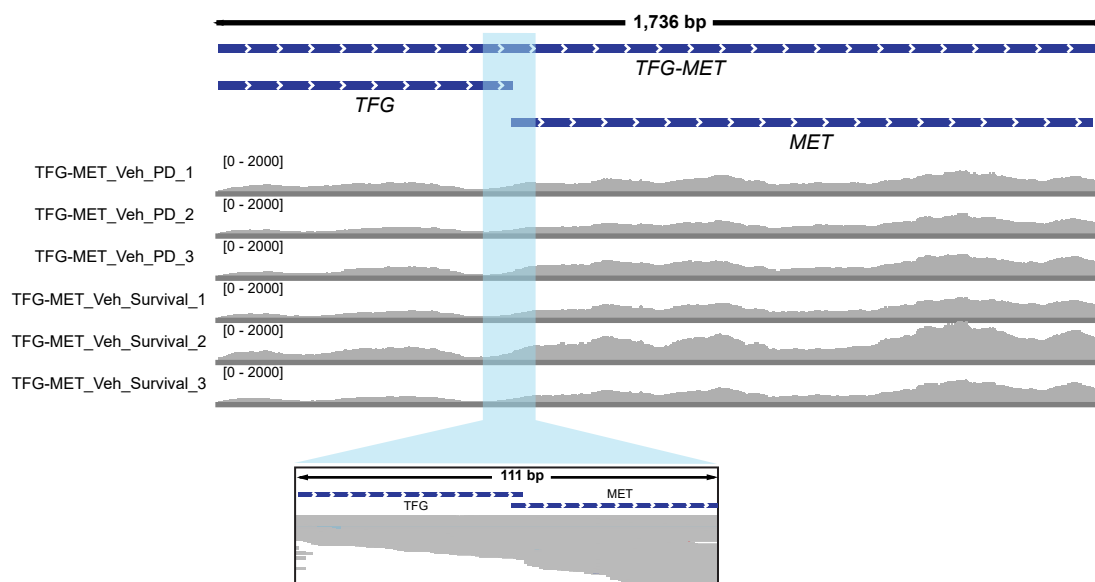

**b**

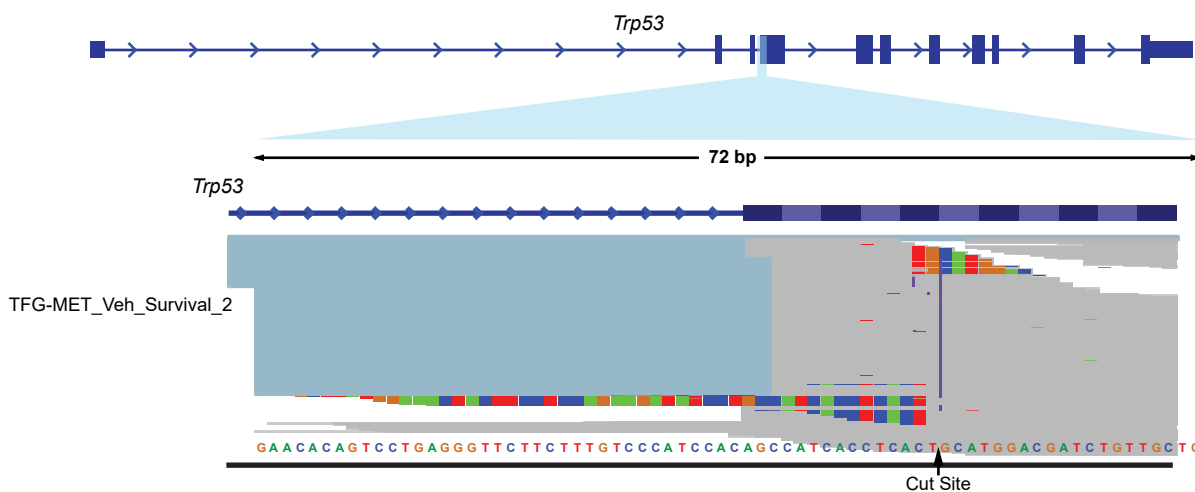

**c**

RNAseq analyses of preclinical allograft trial

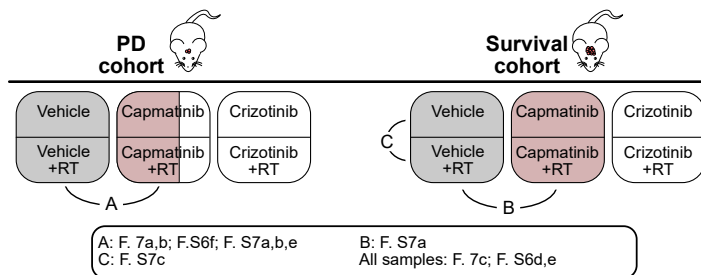

**d**

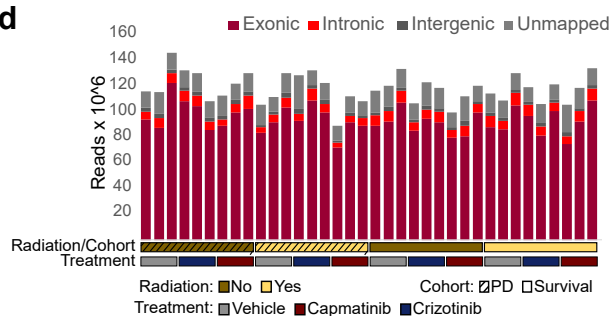

**e**

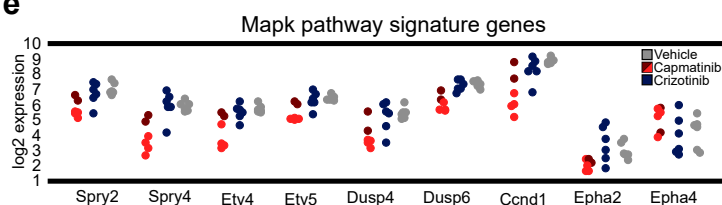

**f**

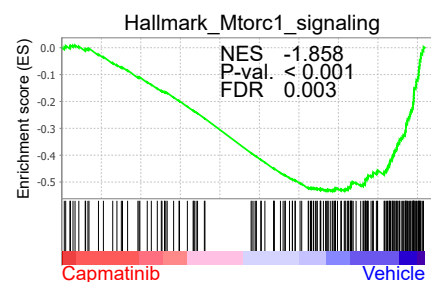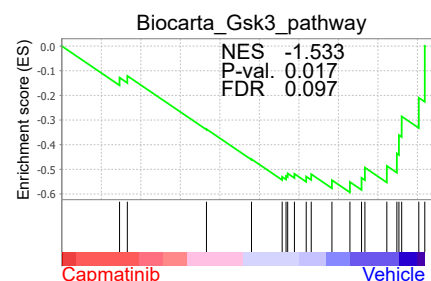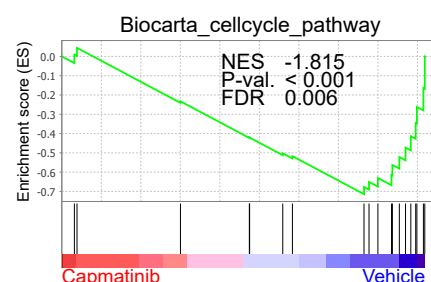

Supplement: Supplementary file 11 — Additional file 11: Supplementary Fig.7. Molecular effects of capmatinib and radiation identified by RNAseq of murine tumors. a, RNAseq coverage from 6 TFG-MET allograft tumors from both the PD and survival cohorts for the TFG-MET fusion construct, notably displaying reads spanning the TFG/MET junction in a representative tumor. b, RNAseq alignments from a representative TFG-MET allograft tumor at the Trp53 locus. The CRISPR-targeted cut site is indicated, at which a 1 bp insertion was observed across tumors. c, Schematic indicating the analyzed groups and the comparisons performed in this figure, Figure 6 and in Extended data Fig.7. The short-term capmatinib-treatment most prominently affected 4 out of 6 analyzed tumors, which were used for comparisons “A”. d, Bar chart indicating a homogenous distribution of reads between all analyzed samples. e, Expression of Mapk pathway signature genes in all samples of both cohorts. Tumors were grouped according to the indicated treatments, irrespective of RT administration. f, Gene set enrichment analyses of the indicated gene sets between vehicle- and capmatinib-treated tumors. Depicted pathways were significantly downregulated after capmatinib treatment. [file 12943_2024_2027_MOESM11_ESM.pdf]

Supplementary Fig. 8

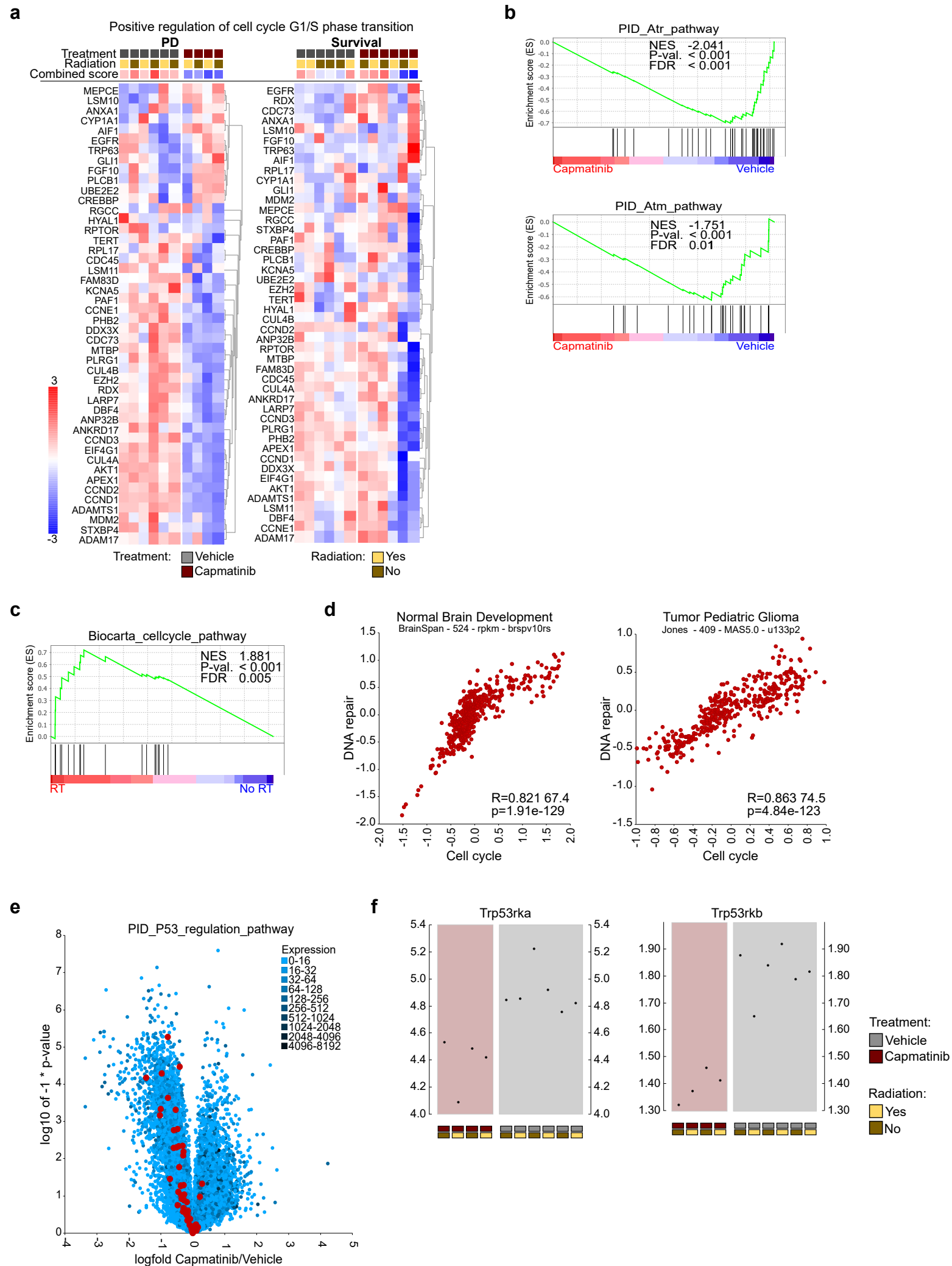

Supplement: Supplementary file 12 — Additional file 12: Supplementary Fig.8. Further molecular data of murine tumors and reference cohorts. a, Cohort-specific heatmaps of genes forming the “POSITIVE REGULATION OF CELL CYCLE G1/S PHASE” geneset (baderlab go 2019). While most analyzed genes are downregulated upon capmatinib treatment in the PD cohort, their expression is tumor-specific and highly heterogenous in the Survival cohort. b, Gene set enrichment analyses of the indicated gene sets between vehicle- and capmatinib-treated tumors of the PD cohort. The Atr- and Atm-pathways are downregulated after capmatinib treatment. c, Gene set enrichment analysis of the indicated gene set between irradiated and non- irradiated tumors of the Survival cohort. Expression of genes involved in cell cycle progression is elevated following RT. d, Correlation between total expression scores of the genesets “DNA REPAIR_7” and “CELL CYCLE_7” (baderlab pathways 2019) amongst all samples in the two depicted datasets. e, Volcano plot indicating all differentially expressed genes between capmatinib- and vehicle-treated allograft tumors. Each dot represents one gene. Red dots (“PID_P53_REGULATION_PATHWAY_3” genes (baderlab pathways 2019)) indicate that most genes involved in regulation of Tp53 signaling are downregulated following capmatinib treatment. f, Expression of Trp53rka and Trp53rkb in indicated tumors of the PD cohort. Both genes are downregulated after capmatinib treatment. [file 12943_2024_2027_MOESM12_ESM.pdf]

# Supplementary Fig. 9

a

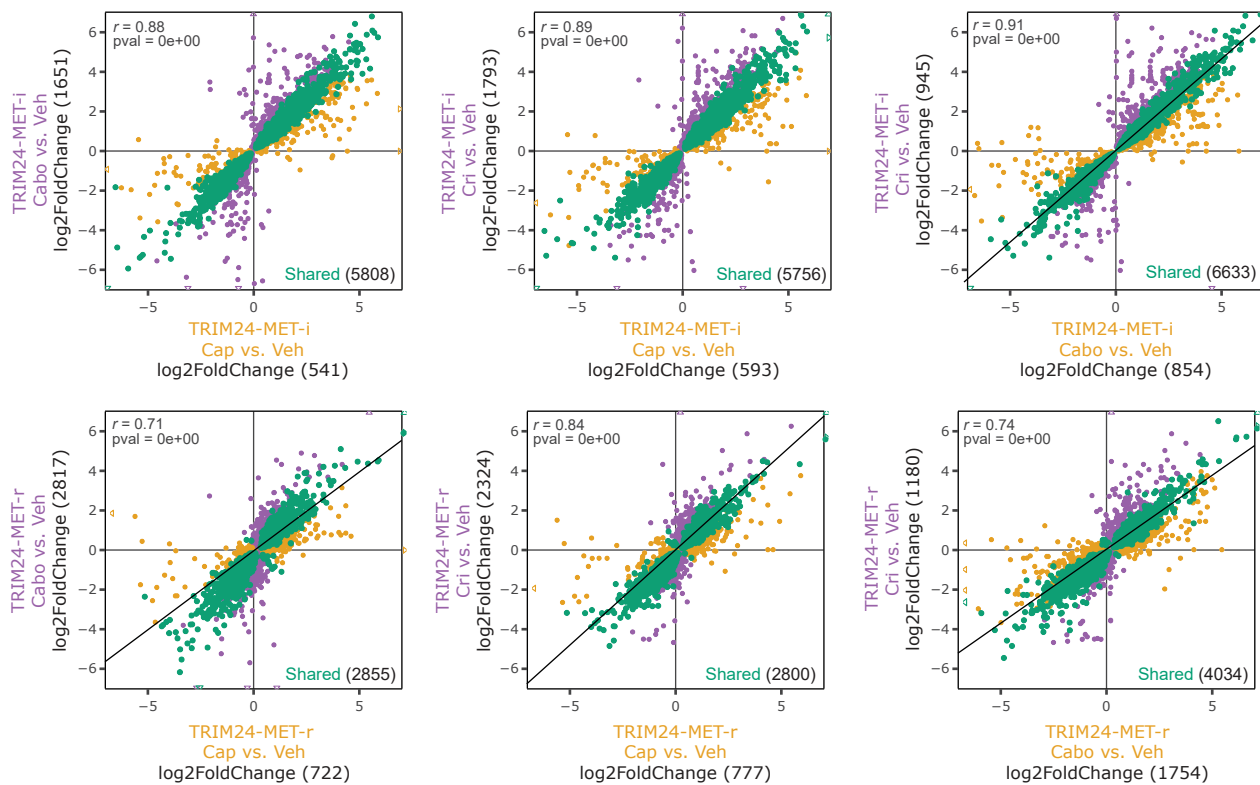

b

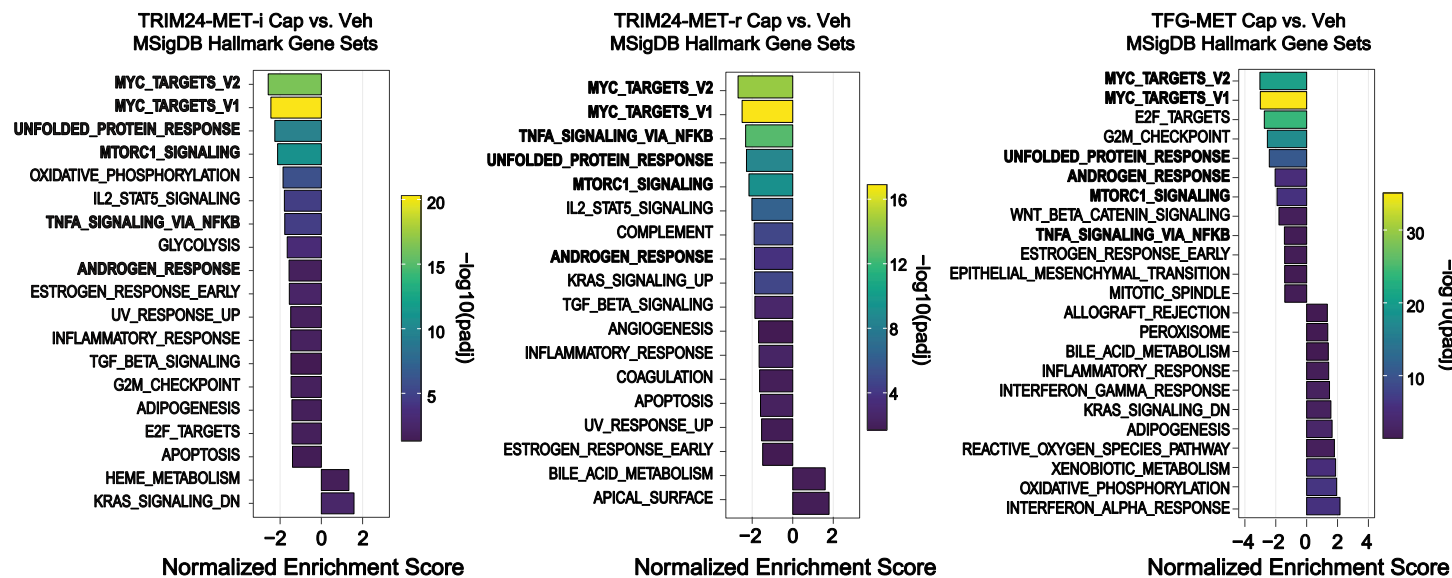

c

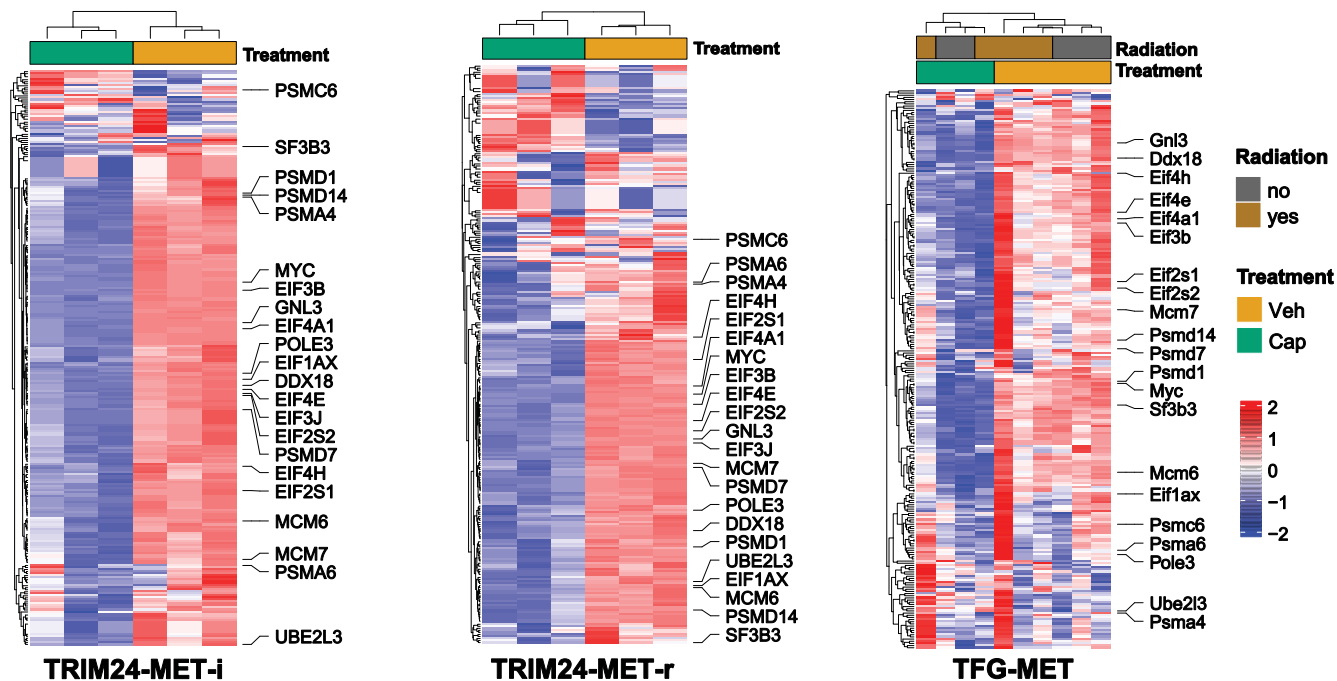

Supplement: Supplementary file 15 — Additional file 15: Supplementary Fig. 9. RNAseq of in vitro and in vivo capmatinib-treated MET fusion models identifies common transcriptomic targets. a, Scatter plots comparing differential gene expression analysis results from TRIM24-MET-i cells (upper panels) or TRIM24-MET-r cells (lower panels) treated with the depicted MET inhibitors in vitro (Cap - capmatinib; Cabo - cabozantinib; Cri – crizotinib; Veh - DMSO). Significant differentially expressed genes (adj. p < 0.05) for the x-axis comparison are colored orange, while those significantly differentially expressed in the y-axis comparison are colored purple. Green points are genes significantly differentially expressed in both comparisons. The number of unique and shared differentially expressed genes between each comparison are shown in parentheses. A linear regression line is depicted along with Pearson’s correlation coefficient (r) and its associated p-value. Open triangles indicate genes beyond the axis-limits. b, Bar plots showing significant (adj. p < 0.05) GSEA results for MSigDB Hallmark genesets for indicated cells and treatment comparisons (TRIM-MET cells were treated in vitro, TFG-MET cells in vivo). Common negatively enriched genesets between all three comparisons are in bold. c, Heatmaps showing expression of Hallmark_Myc_Target_v1/2 genes for indicated cells and treatment comparisons (TRIM-MET cells were treated in vitro, TFG-MET cells in vivo). Select leading edge genes from GSEA are labeled. [file 12943_2024_2027_MOESM15_ESM.pdf]

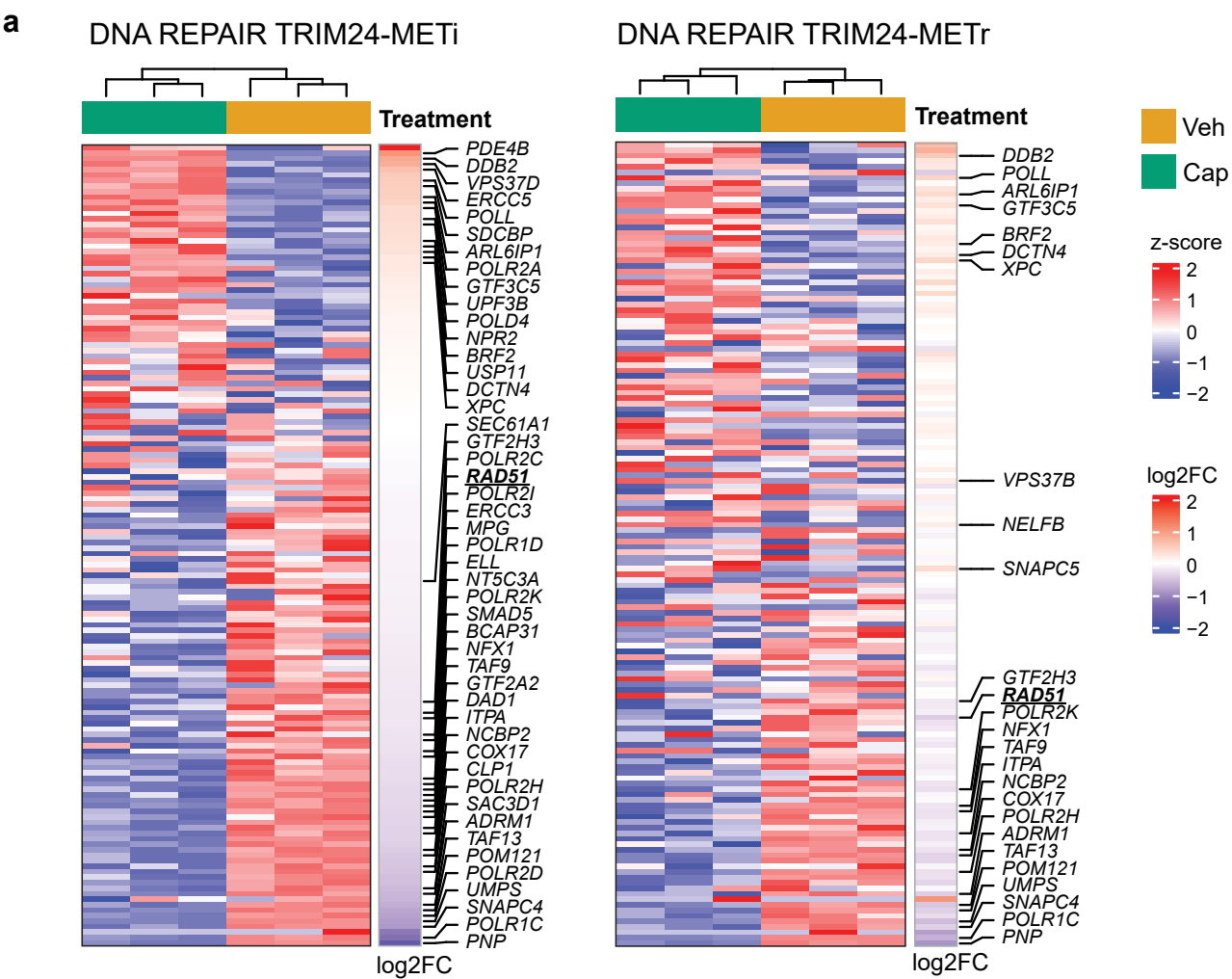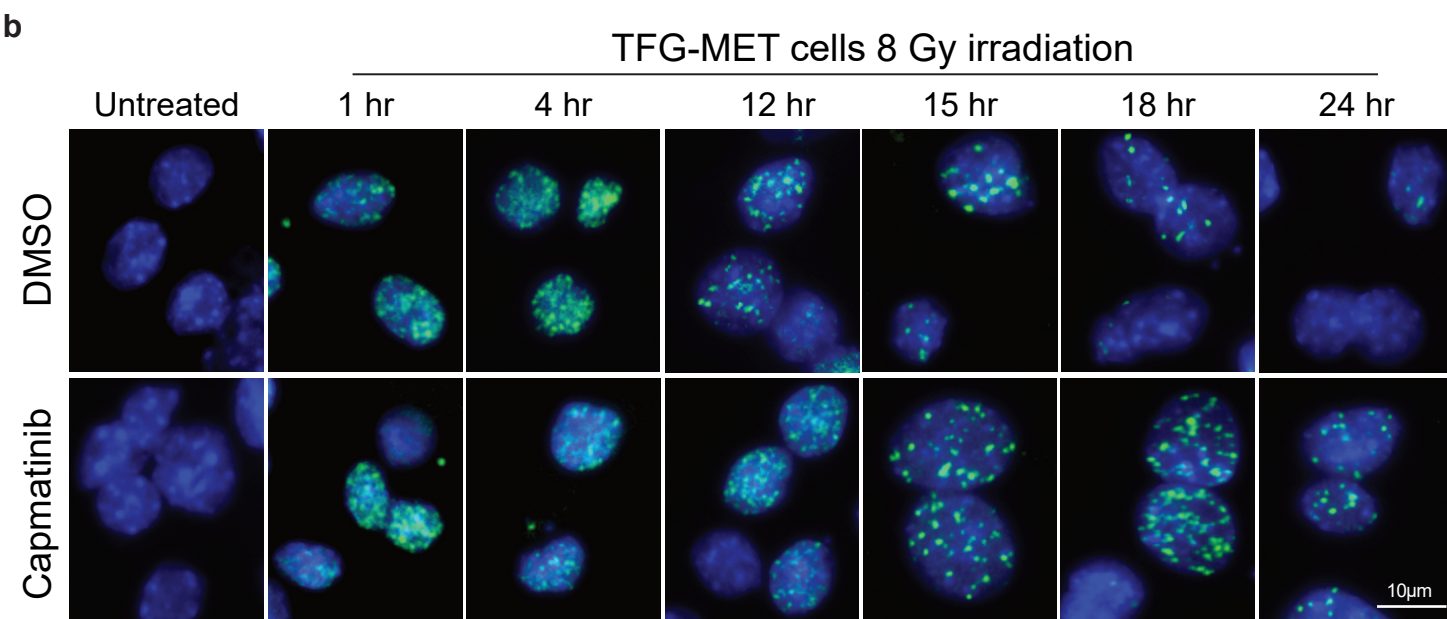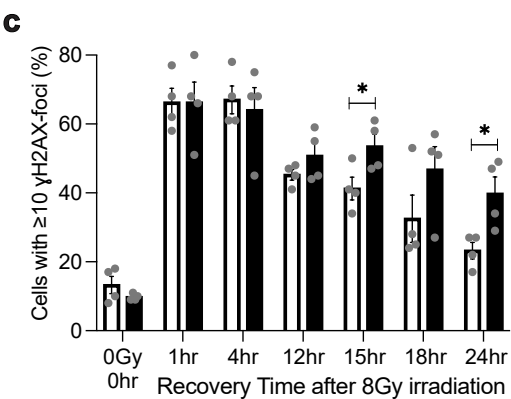

Supplement: Supplementary file 20 — Additional file 20: Supplementary Fig.10. Capmatinib treatment potentiates radiation-induced DNA damage.a, Heatmaps showing expression of Hallmark_DNA_Repair genes (n=146) for in vitro capmatinib treatments in cell lines derived from TRIM24-MET fusion tumors as compared to a DMSO vehicle control. Differentially expressed (p. adj < 0.05) genes in each comparison are labeled, along with RAD51 (bold and underlined). b, γH2AX-immunofluorescence staining of TFG-MET cells at different recovery timepoints following 8Gy-irradiation. Capmatinib (Cap)-treated cells display significantly higher levels of γH2AX compared to DMSO-treated (DMSO) cells. c, Quantification of γH2AX-foci in TFG-MET cells. The percentage of cells with ≥10 γH2AX-foci is significantly higher in capmatinib-treated cells (black bar) compared to DMSO-treated cells (white bar) at various time points following irradiation. Error bars display standard error of mean, statistical significance determined using t-test analysis, *;p<0.05. Scale bar is 10µm. [file 12943_2024_2027_MOESM20_ESM.pdf]
