# Supplementary material for: Capmatinib is an effective treatment for MET-fusion driven pediatric high-grade glioma and synergizes with radiotherapy
Source: Mol Cancer. 2024 Jun 7;23:123. doi: 10.1186/s12943-024-02027-6 (PMC11157767; doi:10.1186/s12943-024-02027-6)

Supplementary Fig. 1

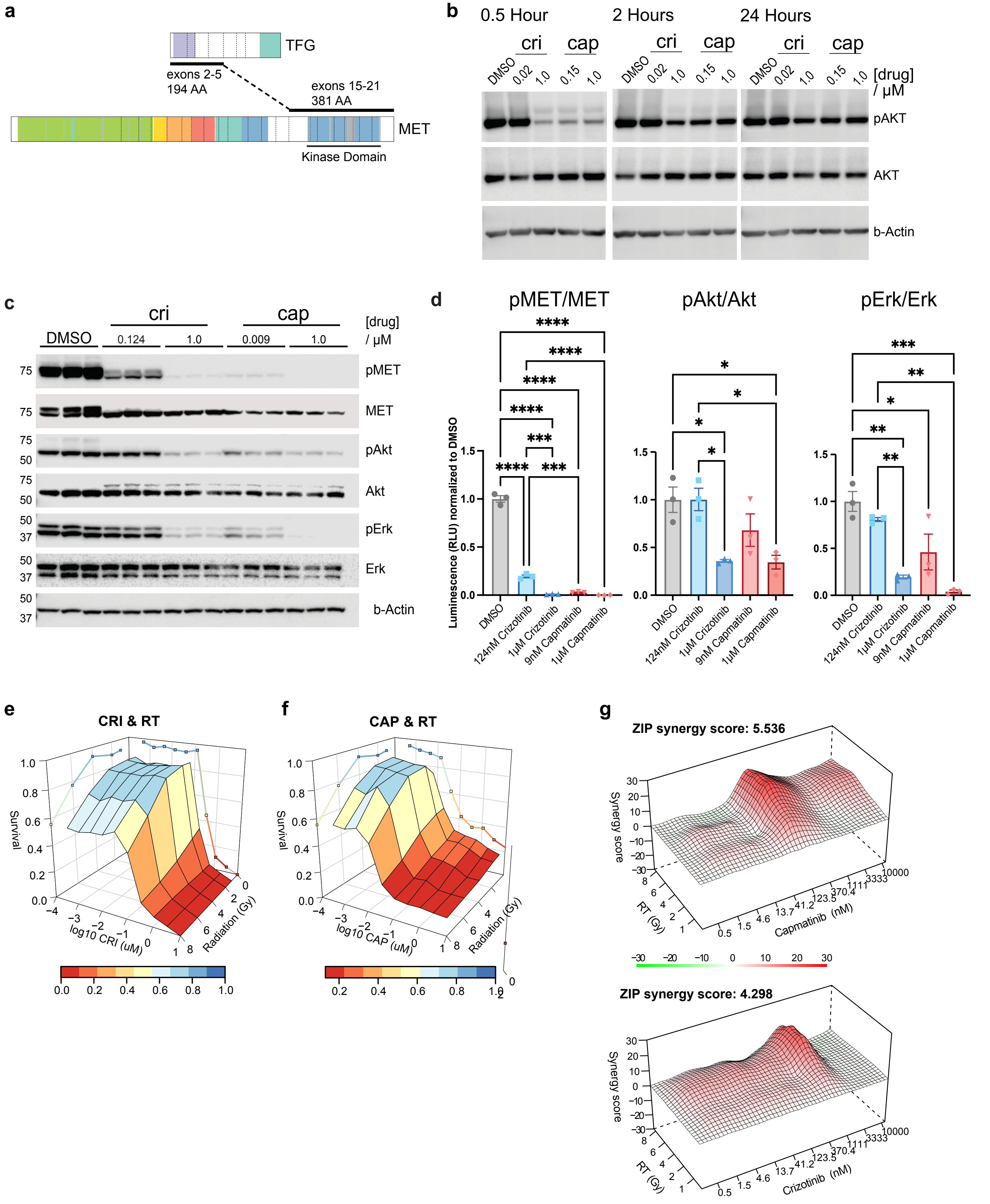

Supplement: Supplementary file 2 — Additional file 2: Supplementary Fig.1. Capmatinib potentiates RT in vitro and in vivo. a, Schematic displaying the TFG-MET fusion gene and protein structure for the construct used for in utero electroporation. b, Western blot of (phospho-)Akt in cultured, murine tumor cells after different time points of crizotinib (cri) or capmatinib (cap) treatment at the indicated concentrations. The results show a quick and temporary inhibition of Akt by both compounds. c, Western blot of phosphorylated and total MET, Akt and Erk, and β-Actin, in cultured murine TFG-MET tumor cells after 2 hours of DMSO control, crizotinib (cri) or capmatinib (cap) incubation at the IC50 for each drug (124nM crizotinib and 9nM capmatinib), and a positive control concentration of 1µM. d, Quantification of luminescence signal of western blots in panel B normalized to the respective DMSO control. Each dot represents an individual replicate. Error bars display standard error of the mean. Statistical significance was determined using a One-Way ANOVA followed by Tukey’s multiple comparisons test (*p<0.05,**p<0.01, ***p<0.001, ****p<0.0001). e,f, 3D illustrations of cell numbers 7 days after treatment with crizotinib and RT (D) or capmatinib and RT (E), respectively. Compound concentrations comprised 10µM and 9 serial 1:3 dilutions in 4 replicates each and radiation doses ranged from 0Gy – 8Gy. Cell numbers were determined with the CellTiter-Glo Assay. g, 3D illustrations of ZIP synergy scores 7 days after treatment with crizotinib and RT (lower panel) or capmatinib and RT (upper panel), respectively. Tested compound concentrations comprised 10 µM and 9 serial 1:3 dilutions in 4 replicates each. ZIP synergy scores were calculated based on cell numbers according to the CellTiter-Glo Assay by using the synergyfinder tool. [file 12943_2024_2027_MOESM2_ESM.pdf]
